# Local adaptation can cause both peaks and troughs in nucleotide diversity within populations

**DOI:** 10.1093/g3journal/jkae225

**Published:** 2024-09-18

**Authors:** Russ J Jasper, Sam Yeaman

**Affiliations:** Department of Biological Sciences, University of Calgary, Calgary, AB, Canada T2N 1N4; Institute of Ecology and Evolution, Department of Biology, University of Bern, 3012 Bern, Switzerland; Swiss Institute of Bioinformatics, 1015 Lausanne, Switzerland; Department of Biological Sciences, University of Calgary, Calgary, AB, Canada T2N 1N4

**Keywords:** local adaptation, divergent selection, nucleotide diversity, migration–selection balance, linkage

## Abstract

The amount of standing variation present within populations is a fundamental quantity of interest in population genetics, commonly represented by calculating the average number of differences between pairs of nucleotide sequences (nucleotide diversity, π). It is well understood that both background and positive selection can cause reductions in nucleotide diversity, but less clear how local adaptation affects it. Depending on the assumptions and parameters, some theoretical studies have emphasized how local adaptation can reduce nucleotide diversity, while others have shown that it can increase it. Here, we explore how local adaptation shapes genome-wide patterns in within-population nucleotide diversity, extending previous work to study the effects of polygenic adaptation, genotypic redundancy, and population structure. We show that local adaptation produces two very different patterns depending on the relative strengths of migration and selection, either markedly decreasing or increasing within-population diversity at linked sites at equilibrium. At low migration, regions of depleted diversity can extend large distances from the causal locus, with substantially more diversity eroded than expected with background selection. With higher migration, peaks occur over much smaller genomic distances but with much larger magnitude changes in diversity. Across spatially extended environmental gradients, both patterns can be found within a single species, with increases in diversity at the center of the range and decreases towards the periphery. Our results demonstrate that there is no universal diagnostic signature of local adaptation based on within-population nucleotide diversity, so it will not be broadly useful for explaining increased F_ST_. However, given that neither background nor positive selection inflate diversity, when peaks are found they suggest local adaptation may be acting on a causal allele in the region.

## Introduction

Understanding how evolutionary processes shape genetic variation is crucial for interpreting patterns across the genome. The number of pairwise differences between nucleotide sequences, the nucleotide diversity (π), is widely used to infer effective population size (Wright 1931; Wang *et al*. 2016), study the signature of selective sweeps (Booker *et al*. 2017), and test theories about the maintenance of variation (Buffalo 2021). As a measure of within-population variation, it is also related to F_ST_, an index used to study patterns of genetic differentiation among populations, as compared with the genetic variation found within populations (Wright 1949). Early genome scan studies identified outlier peaks in F_ST_ as putative indicators of locally adapted loci (reviewed in Nosil *et al*. 2009; Via 2009), because strong selection is expected to drive high differentiation in allele frequency at selected loci (Lewontin and Krakauer 1973) and linked neutral sites (Charlesworth *et al*. 1997; Feder and Nosil 2010). Subsequent reinterpretation of these patterns, however, suggested that outlier peaks in F_ST_ could also be generated by reductions in within-population diversity driven by “linked selection” (Noor and Bennett 2009; Cruickshank and Hahn 2014), either as a result of recent selective sweeps (Smith and Haigh 1974; Kaplan *et al*. 1989; Stephan *et al*. 1992; Braverman *et al*. 1995; Gillespie 1997; 2000; 2001) or background selection (Charlesworth *et al*. 1993; Charlesworth 1994; Hudson and Kaplan 1995; Gillespie 1997). Furthermore, some studies have demonstrated that genome-wide patterns in F_ST_ tend to be inversely correlated with both recombination rate and nucleotide diversity, suggesting that the search for the causal loci driving local adaptation may be obfuscated by the recombination and/or diversity landscapes across the genome (Burri *et al*. 2015; Vijay *et al*. 2017; Irwin *et al*. 2018).

In light of these studies, it is now well recognized that both hard sweeps and background selection have the potential to reduce genetic variation at linked sites (Booker *et al*. 2021), and they are now commonly invoked to explain signatures where a relative local reduction in nucleotide diversity is found in some areas of the genome along with elevated F_ST_. Complicating the picture somewhat, recent theoretical work has suggested that background selection likely only very minimally affects F_ST_ and that any detected peaks are therefore unlikely to be generated solely as a function of reduced diversity due to background selection (Matthey-Doret and Whitlock 2019). Still other studies have shown that uniform positive selection can generate high among-population variation as a consequence of incomplete sweeps or recombination during a sweep, which may be sufficient to explain observed genome-wide patterns of F_ST_ in many cases (Bierne 2010; Booker *et al*. 2021).

Regardless of what drives genome-wide patterns of F_ST_, it remains that local adaptation *does* often occur (Hedrick *et al*. 1976; Linhart and Grant 1996; Hereford 2009), and as such, some signatures of elevated F_ST_ may also be expected in species with local adaptation. Within-population nucleotide diversity (hereafter: π_w_) is now commonly used to infer the possible activity of background or positive selection at linked sites, and therefore also used to inform whether observed F_ST_ values might indeed be driven by local adaptation. However, it is not immediately clear how local adaptation should affect π_w_ and whether the expectation should differ from background or positive selection. It is therefore important to understand how local adaptation affects π_w_, especially as it pertains to interpreting genome scan results.

For an unlinked neutral locus in an island model, expected π_w_ is 4·*N*_e_·*μ* when migration rate is 0 and 4·*d*·*N*_e_·*μ* when *m* > 0 (where *N*_e_ is effective population size, *μ* is the per locus mutation rate, *m* is migration rate, and *d* is the number of demes; Nordborg 1997; Wakeley 2009). When a neutral locus is linked with a locally adapted locus and migration is rare, nucleotide diversity will be depleted at the neutral site (Nordborg 1997). Nordborg (1997) likened the effect of migration–selection balance in this range of parameter space to that of background selection, which also decreases π_w_ at linked sites (also see Aeschbacher and Bürger 2014; Fig. 8). It is less clear, however, what happens to π_w_ as migration rate increases, and the above models make assumptions that limit their applicability to high migration. It is well accepted that strong balancing selection increases total nucleotide diversity at linked sites (Hudson and Kaplan 1988; Barton and Navarro 2002; Charlesworth 2006), and divergent selection with high migration would therefore have similar effects (Nordborg and Innan 2003). It is unclear, however, how such diversity would be partitioned within vs among populations and how this would change with migration–selection balance. The set of individuals with a given divergently selected haplotype can be considered analogous to a population, and we would expect reduced diversity around a selected site within this set, and increased diversity among sets. However, while selection acts to increase the assortment of locally adapted haplotypes to the population where they are favored, migration mixes haplotypes and inflates diversity, and as such, increased π_w_ might be expected. Indeed, Charlesworth *et al*. (1997) showed an increase in π_w_ near the locally adapted locus for a model that also included background selection (their Fig. 7A), and for an analytical model without background selection (their Table 1). More recently, Sakamoto and Innan (2019) analyzed a two-population model and found a small peak in π_w_ around the locally adapted locus, but focused more of their analysis on the decrease in π_w_ around the selected site that occurs during the initial establishment of a locally adapted polymorphism. As most studies on the effect of migration–selection balance on π_w_ at linked sites have used relatively simple genetic architectures and patterns of population structure, further consideration of this question is necessary.

**Table 1.** Model parameters and associated values.

Model	Number of Adaptive Loci	Genotypic Redundancy	Adaptive Allele Effect Size	Adaptive Locus Mutation Rate	Mutation Model	Strength of Selection on Genotype (*V*_S_)	Number of Patches (*d*)	Phenotypic Optima (*θ*; by patch)	Patch Size (*N*)
Single-Locus, Two-Patch	1	Nonredundant	Sampled from N(0,1)	10^−5^, 10^−2^/N	Continuum of Alleles	2, 5, 10, 25, 100, 10^9^	2	−1, + 1	500, 1000, 2000, 10,000
MultiLocus, Nonredundant, Two-Patch	1, 2, 4, 10, 20, 50, 100	Nonredundant	$\pm \frac{1}{2l}$ (*l* = number of adaptive loci)	10^−5^	House of Cards	5	2	−1, + 1	1000
MultiLocus, Redundant, Two-Patch	4, 10, 20, 50, 100	Redundant	±$\frac{1}{4}$	10^−5^, 10^−4^	House of Cards	5	2	−1, + 1 (divergent); +1, + 1 (uniform)	1000
Single-Locus, Ten-Patch	1	Nonredundant	Sampled from N(0,1)	10^−5^	Continuum of Alleles	5	10	−1, −$\frac{7}{9},\;$−$\frac{5}{9},$ −$\frac{3}{9},\ldots ,+1$	1000

In the present study, we describe how local adaptation over heterogeneous landscapes shapes patterns in nucleotide diversity (both π_w_ and d_xy_) and F_ST_ at the neutral regions flanking a selected locus over a wide range of migration–selection parameter space. We begin by using individual-based simulations of two-patch models with a single selected locus, then explore models with polygenic adaptive traits and varying degrees of genotypic redundancy, and lastly, investigate more complex patterns of population structure by exploring a ten-patch stepping stone model. We show that both troughs and peaks in π_w_ may be expected around a locally adapted locus, depending on the parameters involved.

## Materials and methods

To understand how local adaptation over heterogeneous landscapes shapes patterns in nucleotide diversity we performed simulations using two different landscape models: a two-patch model (with and without genotypic redundancy) and a linear ten-patch model (Table 1; Supplementary Fig. 1). We performed our simulations with the stochastic, forward-time, individual-based simulation program Nemo, version 2.3.46 (Guillaume and Rougemont 2006). Our simulations followed the Wright–Fisher model with the addition of selection, migration, and mutation. We modeled a single trait under Gaussian stabilizing selection, where the fitness of an individual (*W*) was defined as


$$W(z)\;=\;e^{\frac{-{\left(z\;-\;\right)}^{2}}{2\cdot V_{s}}}$$

Where *z* was an individual's phenotypic value, an additive function of the allele effect size at each locus (i.e. no epistasis or dominance effects on phenotype); *θ* was the optimal phenotypic value of the local patch; and *V*_S_ was the strength of selection on the genotype as described by the variance around the fitness function. Unless otherwise stated, a *V*_S_ of 5 was used. After calculating the base fitness value of each individual within a patch, an individual's fitness was scaled against the mean fitness of the local patch.

Individuals had a single diploid chromosome where each divergently selected locus was symmetrically flanked by 74 neutral loci positioned at distances from 10^−3^ to 10 cM away on a log_10_ scale (Supplementary Figs. 2 & 3). In the case of multiple adaptive loci on a chromosome, each adaptive locus and its 74 flanking neutral loci was separated from the next closest adaptive locus and associated neutral loci by 50 cM, such that one complement of 75 loci was unlinked from any other complement of 75 loci (Supplementary Fig. 3). Neutral loci were diallelic and mutation occurred at a rate of 10^−5^ per locus per generation. Similarly, unless otherwise stated, mutation occurred at the selected loci at a rate of 10^−5^. Simulations were initialized whereby neutral loci were randomly assigned allele values. We acknowledge that maximizing the initial standing variation in this way is not the most biologically realistic scenario, however, the alternative option afforded by Nemo was to initialize simulations with zero standing variation, no more biologically realistic. We show that our results are qualitatively insensitive to the degree of standing variation the populations were initialized with (Supplementary Fig. 4).

Forward migration rates were varied between 10^−5^ and 10^−½^ with four equal increments per order of magnitude, in addition to a migration rate of zero. Unless otherwise stated, each patch was comprised of 1,000 individuals. Each simulation replicate was run for a total of 25·*N*·*d* generations, where *N* was the population size by patch and *d* was the number of patches in the metapopulation. The within- and between-population nucleotide diversity, total metapopulation nucleotide diversity and F_ST_ were then calculated at each locus after 25·*N*·*d* generations, except in the case of the single-locus, two-patch model, where they were iteratively calculated every ½·*N*·*d* generations. After this amount of time, populations had typically approached a steady state, but we note that at very low migration rates, populations approached true equilibrium very slowly (e.g. Supplementary Fig. 5), so we refer to this point as quasi-equilibrium.

### Single-locus, two-patch model

In the single-locus, two-patch model, the adaptive locus was multiallelic and mutation occurred whereby a new allele effect size was drawn from a normal distribution (*μ* = 0, *σ^2^* = 1) and added to the former allele effect size (i.e. continuum of alleles). Two different mutation rates were explored, an unscaled rate independent of population size (10^−5^) and a scaled rate standardized by population size (10^−2^/*N*) (i.e. populations had the same number of mutational events per generation regardless of population size).

### Multiple adaptive loci, two-patch model

In models with polygenic adaptive traits, adaptive loci were diallelic and mutation occurred whereby the original allele would be replaced by the opposite allele (i.e. house of cards). In the genotypically nonredundant model, the allele effect sizes were scaled relative to the number of adaptive loci such that an individual needed to be homozygous for the optimal allele at every locus in order to achieve the optimal phenotype in a given patch. By contrast, in the genotypically redundant model, the allele effect sizes were set to ±0.25 regardless of the number of loci, such that an individual could reach the phenotypic optimum (±1 divergent selection; +1 uniform selection) by being homozygous for the optimal alleles at any two loci.

### Single-Locus, ten-patch model

Our ten-patch model (Supplementary Fig. 1) consisted of demes in a linear conformation and followed the stepping stone migration model (i.e. dispersal was only possible between directly adjacent patches). The phenotypic optimum scaled linearly across the ten patches. The adaptive locus was multiallelic and mutation occurred as in the single-locus, two-patch model, where a new allele effect size was drawn from a normal distribution (*μ* = 0, *σ^2^* = 1) and added to the former allele effect size (i.e. continuum of alleles).

### Study metrics

Nucleotide diversity (π) was calculated as


$$\pi =\underset{ij}{\sum }\;x_{i}x_{j}\pi_{ij}$$

Where *x_i_* and *x_j_* were the respective frequencies of the *i*^th^ and *j*^th^ sequences in a population and *π_ij_* was the number of nucleotide differences between the *i*^th^ and *j*^th^ sequences (Nei and Li 1979).

As well, we report the F_ST_ per locus returned by Nemo, which was calculated using Weir and Cockerham (1984). We regressed the nucleotide diversity or F_ST_ at a neutral locus on its log_10_-transformed distance from an adaptive locus (cM) to quantify the relationship between diversity or F_ST_ and distance from an adaptive locus. As an additional way to explore the effect of selection and linkage on diversity, we compared the levels of nucleotide diversity or F_ST_ at the neutral loci 0.001 cM away from the adaptive locus to those 10 cM away.

In order to study the effect of selection across the chromosome, we calculated the mean level of nucleotide diversity that persisted under strong selection (*V*_S_ of 5) at all neutral loci between 9 and 10 cM away from the adaptive locus, and compared this to the genome-wide background levels of diversity across the chromosome under neutral evolution (*V*_S_ of 10^9^). For this specific analysis we added additional neutral loci to the ends of the chromosome, such that there were 100 loci positioned from 9 to 10 cM away from the adaptive locus, in even steps, at either end of the chromosome. The additional neutral loci were added in order to reduce the noise in our result and did not affect the overarching patterns seen.

We identified peaks in nucleotide diversity as those with a significant slope of diversity by log_10_-transformed distance according to a t-test and at least 25% of neutral loci with diversity levels in excess of 1.1 times the genome-wide background level. To quantify the width of a peak in diversity, we regressed the nucleotide diversity of all neutral loci with levels in excess of the genome-wide background on their distance from the adapted locus; the *x*-intercept of the regression was taken as one half of the width of the peak.

Finally, we estimated pairwise linkage disequilibrium between the adapted locus and each neutral locus across the metapopulation as a whole. Pearson's *r*^2^ was taken as an estimate of linkage disequilibrium.

### Comparison with previous analytic predictions

We compared our simulation results for the π_w_ at a tightly linked locus (10^−3^ cM) to the analytical predictions for the expected within-population heterozygosity from Sakamoto and Innan (2019) equation 25. Where we model Gaussian fitness acting on the phenotype (to facilitate comparison among single- and multilocus traits), Sakamoto and Innan (2019) used selection coefficients acting on an individual locus in each patch (*s_i_*). To compare models, we set each of the *s* terms in equation 25 to match the reduction in fitness for a locally optimal individual moving to the nonoptimal patch (and ignored the effect of dominance), which requires asymmetrical coefficients (*s*_1_ ≠ |*s*_2_|). We also report the comparison between models under symmetrical selection coefficients.

We note that it can be difficult to match simulation results with analytical predictions when very rare events contribute to average behavior. For example, under a model of pure neutrality, the result that mean π_w_ is invariant with migration in a finite island model (Nordborg 1997; Wakeley 2009) occurs because when migration is very low, most replicates have very low nucleotide diversity (near 4*N*_e_*μ*), but in rare cases a recent migrant introduces large amounts of variation (due to high divergence among lineages), such that on average π_w_ = 4*dN*_e_*μ*. As results from simulations take the average across a given set of replicates, the mean π_w_ estimated in simulations can tend towards 4*N*_e_*μ* for neutral loci when migration rates are very low, if the sampled replicates do not happen to include a recent migrant (Supplementary Fig. 5). Thus, simulation results can appear at odds with analytical predictions about mean π_w_, but are representative of the median behavior of π_w_, which is likely more biologically realistic than the arithmetic mean (given that when migration is very low, few if any individual replicates actually have π_w_ = 4*dN*_e_*μ*; Supplementary Fig. 5). This illustrates a discordance that is also encountered in our simulations of selection with linked neutral loci: at low migration rates, taking the average across a large but finite number of replicates may fail to capture the influence of very rare migrants on arithmetic mean π_w_, but do still represent the average behavior of most replicates (which is arguably biologically more realistic).

## Results

### Single-locus, two-patch model

#### Migration–selection balance

**Fig. 1.**
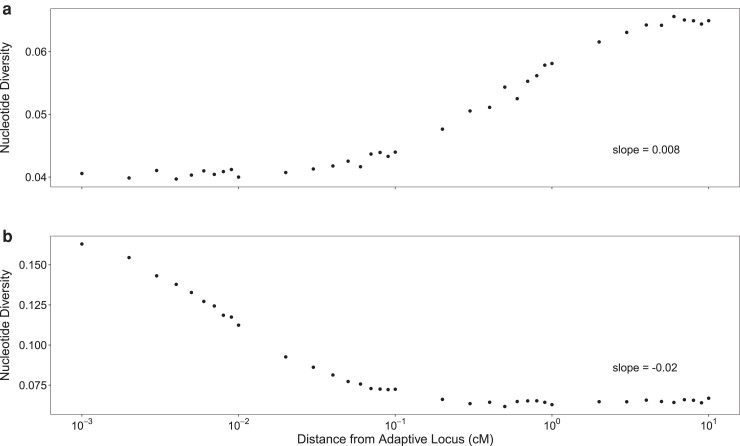
Patterns of π_w_ along the chromosome at neutral loci linked to a single divergently selected locus in two-patch model. Mean diversity is shown for migration rates *m* = 10^−3.5^ (a) and *m* = 10^−1.5^ (b) after 50,000 generations. The magnitude of each diversity-distance slope (π_w_ vs log_10_ cM) is shown in bottom right of each panel. Each patch was comprised of *N* = 1,000 individuals, mutation rate = 10^−5^ per locus, and *V*_S_ = 5.

**Fig. 2.**
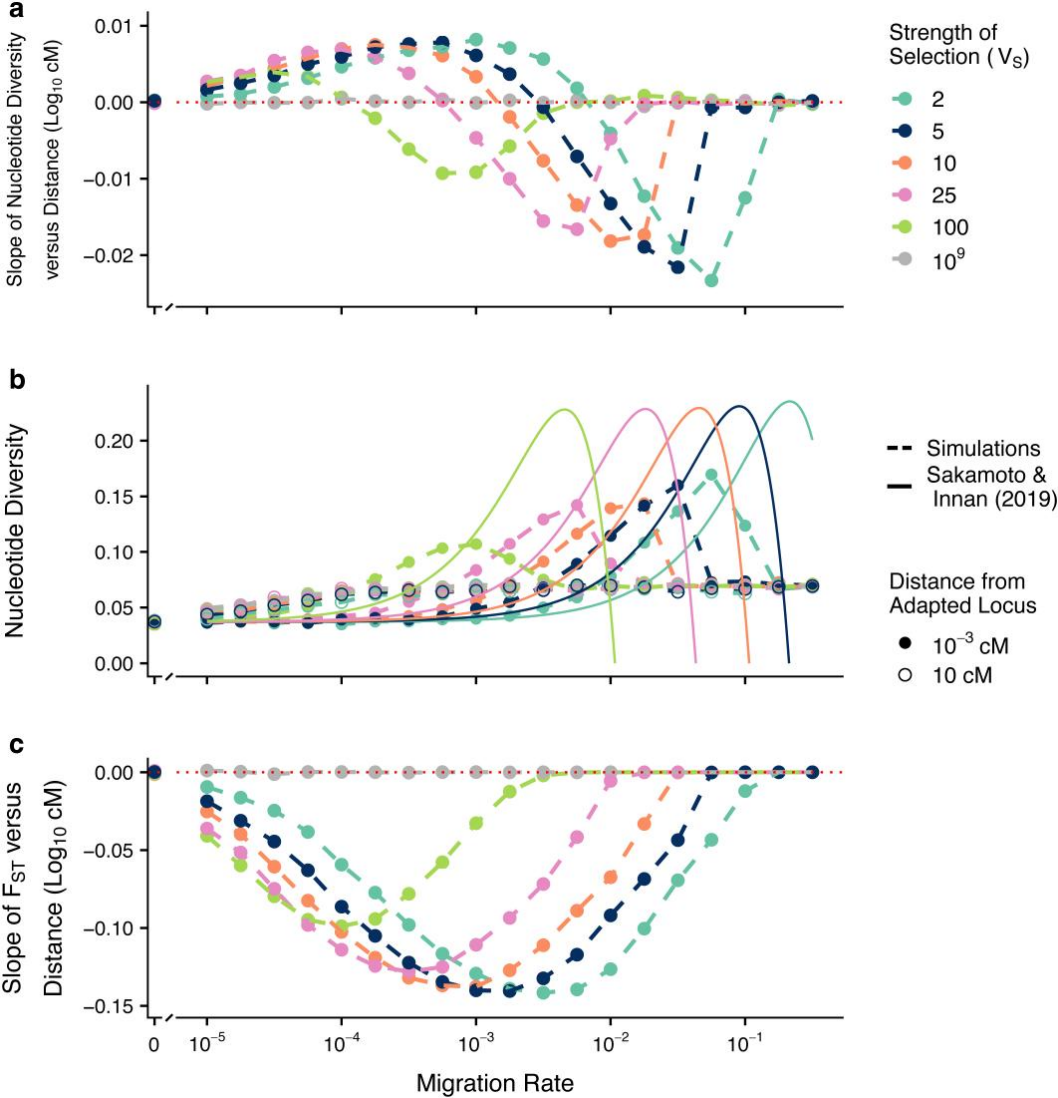
Effect of migration–selection balance on genetic variation at neutral sites linked to a single divergently selected locus in two-patch model. The slope of π_w_ vs distance (log_10_ cM) (a), the π_w_ at the loci closest (10^−3^ cM) and furthest (10 cM) from the locally adapted locus (b), and the slope of F_ST_ vs distance (log_10_ cM) (c) are shown against the log_10_ migration rate after 50,000 generations. Each patch was comprised of *N* = 1,000 individuals and the per locus mutation rate = 10^−5^. Simulation results are shown in dashed lines, analytical predictions from Sakamoto and Innan (2019) equation 25 using asymmetrical selection coefficients are shown in solid lines.

To explore the effect of selection on π_w_ at linked sites, we calculated the slope of the regression of mean π_w_ on the distance from the selected locus, which we will refer to as the diversity-distance-slope (dd-slope). When this slope was positive, π_w_ tended to be substantially depressed near the selected site (as occurs with a selective sweep or background selection), whereas a peak in π_w_ around the selected site was present when the slope was negative (see Fig. 1 for examples). In the single-locus, two-patch model we observed a nonmonotonic relationship between migration rate and the dd-slope, with slopes of zero when migration rate was zero, positive slopes at very low migration rates, and negative slopes at intermediate–high migration rates, with a transition between these opposite patterns at low-intermediate migration rates (Fig. 2a). These patterns can also be seen by contrasting the π_w_ at the neutral loci nearest the adapted locus compared to those furthest away (Fig. 2b). When the dd-slope was positive we found that the π_w_ was substantially depressed across the entire length of the chromosome (10 cM on either side of the selected locus) relative to genome-wide background levels (Supplementary Fig. 6a), whereas when the dd-slope was negative we observed a more restricted effect across the chromosome, with peaks in π_w_ of 0.14–1.18 cM in width beyond background levels (Supplementary Fig. 6b).

In cases where migration was too high to permit the maintenance of local adaptation (e.g. *m* > 10^−1.25^ at *V_S_* = 5; Supplementary Fig. 7), the dd-slope tended to return to 0 (Fig. 2a) and the per locus π_w_ at tightly linked neutral loci declined to approximate the drift expectation, as indicated by the case with *V*s = 10^9^ (gray line, Fig. 2b). Increasing the strength of selection shifted the above-described patterns so that the peaks and transitions occurred at higher rates of migration, and also increased the maximum magnitudes of both the peak positive and peak negative dd-slopes (Fig. 2a-b).

Additionally, we used the same approach to examine patterns in total metapopulation nucleotide diversity, d_xy_ and F_ST_. As might be expected from previous theoretical work (Charlesworth *et al*. 1997; Sakamoto and Innan 2019), the between-population dd-slope (Supplementary Fig. 8) and the slope of F_ST_ by distance (Fig. 2c) were always negative and reached a maximum magnitude at intermediate migration rates, as these conditions maximized the difference between the d_xy_ or F_ST_ at the selected locus and the same metric at unlinked neutral loci.

We found high qualitative concordance between our results for the π_w_ at a tightly linked locus (10^−3^ cM) and Sakamoto and Innan's (2019) analytical prediction for the expected heterozygosity with both asymmetrical (Fig. 2b) and symmetrical nonzero selection coefficients (Supplementary Fig. 9). In our simulation results, however, we did find a strong effect of the strength of selection on the maximum magnitude of the peaks in diversity observed over high migration rates. Conversely, the magnitude of the peaks in expected heterozygosity predicted from Sakamoto and Innan (2019) appear relatively insensitive to the strength of selection.

#### Effects of population size

**Fig. 3.**
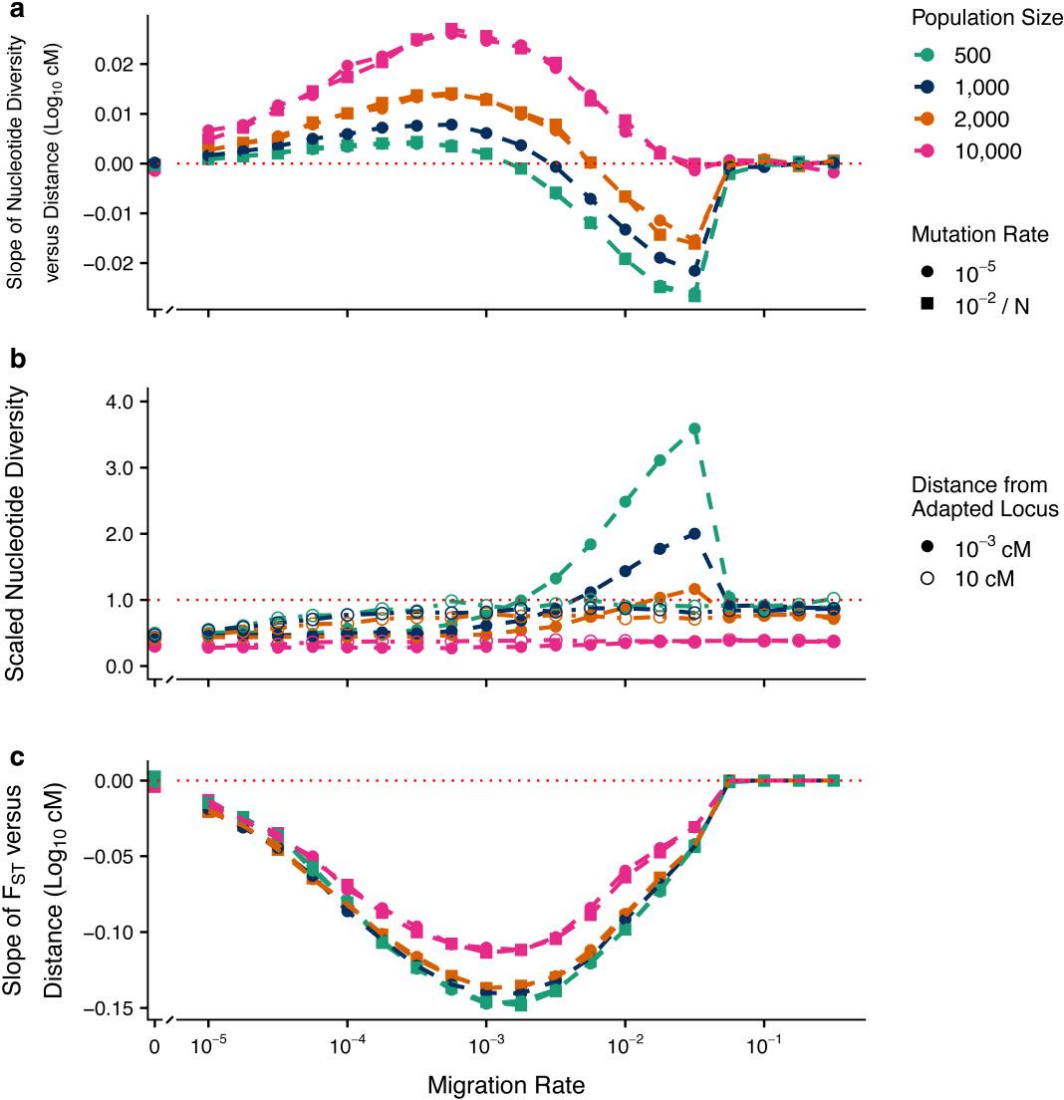
Effect of migration–selection balance and population size on genetic variation at neutral sites linked to a single divergently selected locus. The mutation rate at the selected locus was 10^−5^ (unscaled) or 10^−2^/*N* (scaled), the mutation rate at neutral loci was 10^−5^ per locus, and *V*_S_ = 5. Panels (a) and (c) are as described in Fig. 2; panel (b) shows the π_w_ scaled by the neutral expectation at *m* > 0 (4·*d*·*N*·*μ*).

We investigated how altering the population size of each patch affected the patterns in π_w_ and F_ST_ described above. Broadly speaking, increasing the population size increased the value of both the dd-slopes and the slopes of F_ST_ by genetic distance over all migration rates below the critical rate (Fig. 3, a and c). Over intermediate–high migration rates, increasing the patch size to 10,000 eliminated the negative dd-slope trend (Fig. 3a) and any peaks in π_w_ beyond the neutral expectation (Fig. 3b) seen with smaller population sizes. Across these intermediate–high migration rates, the linkage disequilibrium between neutral loci and the locally adapted locus decayed much more rapidly with larger population sizes (Supplementary Fig. 10). Finally, there was little noticeable effect of scaling the mutation rate by population size (Fig. 3, a and c).

#### Patterns through time

To explore how patterns in π_w_ and F_ST_ might change with time, we examined the slopes at 1000-generation intervals. Both the dd-slopes and the slopes of F_ST_ by distance consistently decreased with time until reaching their respective quasi-equilibrium values (Supplementary Fig. 11, a and c). Additionally, the per locus π_w_ steadily decreased with time until equilibrating, where the loci closest to the locally adapted locus reached quasi-equilibrium earlier and the loci furthest away later (Supplementary Fig. 11b).

### Effects of multiple adaptive loci and genotypic redundancy

**Fig. 4.**
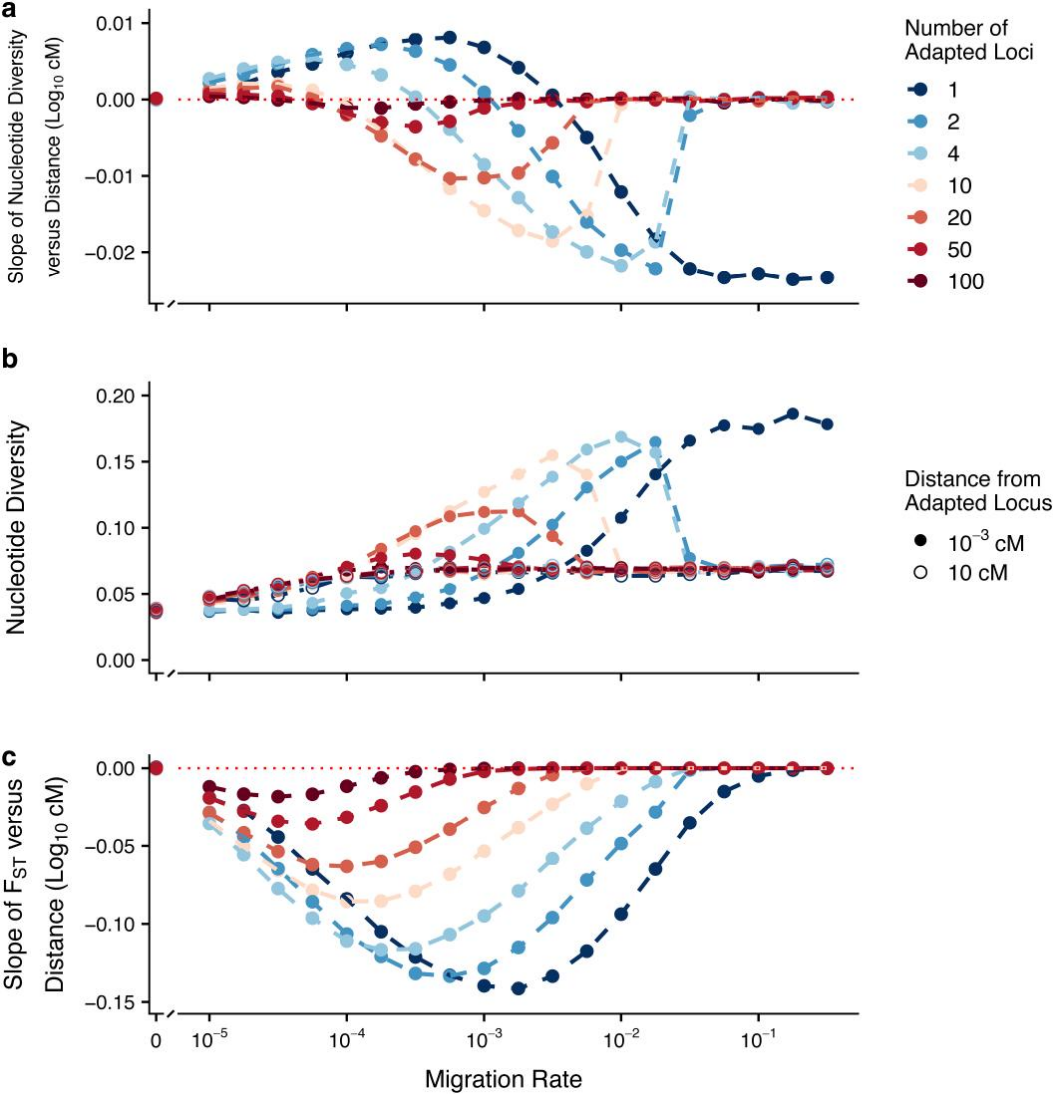
Effect of migration–selection balance on genetic variation at linked neutral sites for a quantitative trait with different numbers of loci and no genotypic redundancy. Allele effect sizes were scaled by the number of adaptive loci, such that an individual could only reach the optimum in a given patch by being homozygous for the optimal allele at each locus. Each patch was comprised of *N* = 1,000 individuals, mutation rate = 10^−5^ per locus, and *V*_S_ = 5. Panels (a–c) are as described in Fig. 2.

**Fig. 5.**
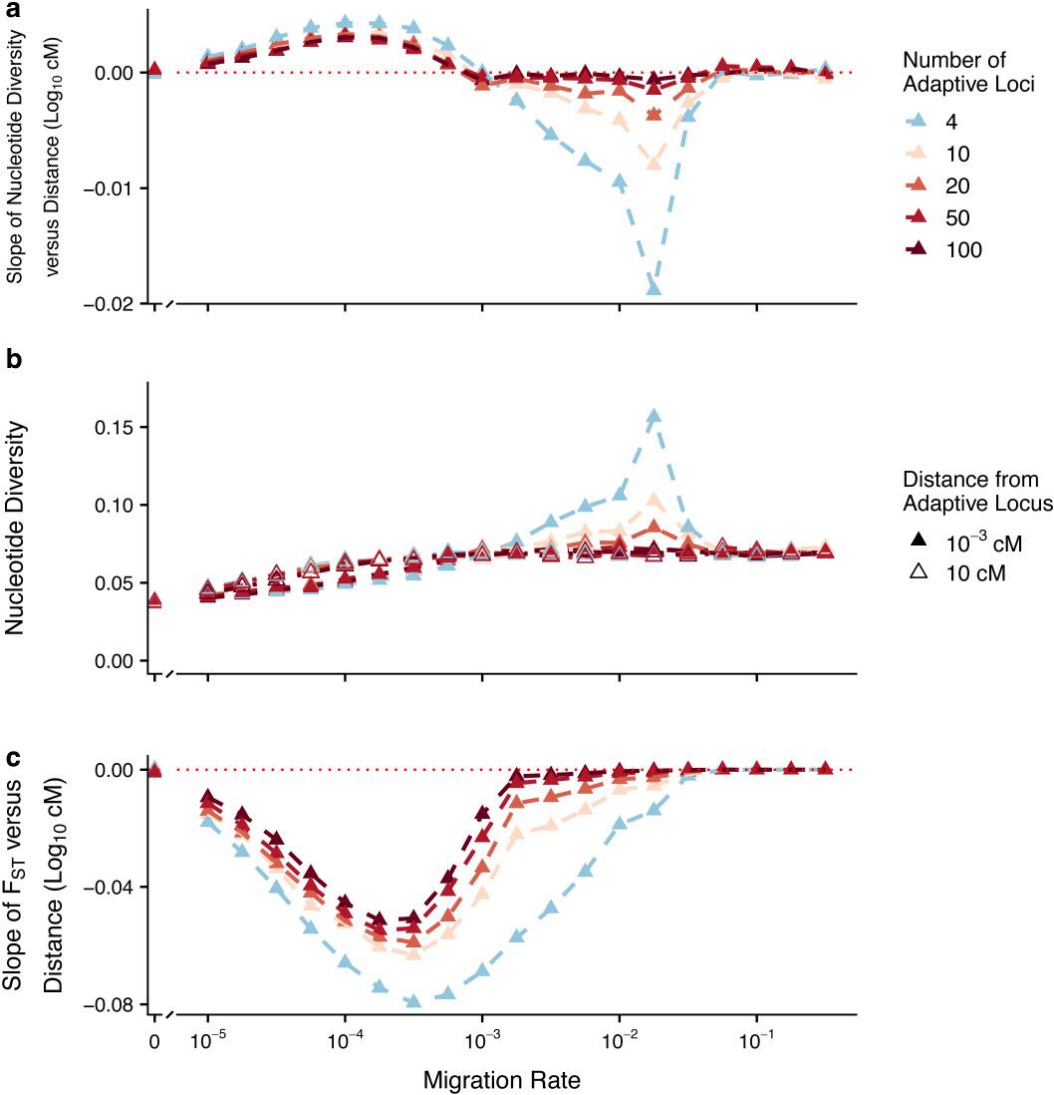
Effect of migration–selection balance on genetic variation at linked neutral sites for a quantitative trait with different numbers of loci and variable levels of genotypic redundancy. Allele effect sizes were ±0.25, such that an individual could reach the optimum in a patch (±1) by being homozygous for the optimal allele at 2 loci. Each patch was comprised of *N* = 1,000 individuals, mutation rate = 10^−5^ per locus, and *V*_S_ = 5. Panels (a–c) are as described in Fig. 2.

When there was no genotypic redundancy (i.e. when mutations at all loci were needed to yield a locally optimal phenotype), an increase in the number of adaptive loci corresponded to a decrease in the net effect of selection on each individual locus, as each locus had a smaller allele effect size. Thus, the effect of increasing the number of loci (Fig. 4) closely resembled the effect of reducing the strength of selection observed in the single-locus model (Fig. 2). In contrast, when there was genotypic redundancy in the trait (i.e. more loci than the number of mutations needed to reach the local optimum) and each adaptive locus had the same effect size regardless of the total number of loci involved, increasing the number of loci did not shift the patterns of dd-slope with migration through the parameter space (Fig. 5).

Across low migration rates where we found reduced π_w_ around the focal site, we observed an interaction between the number of adaptive loci contributing to a trait and whether or not there was genotypic redundancy (Fig. 4 & 5). In the case with no redundancy, the relationship between dd-slope and migration attenuated with an increasing number of loci, with the transition point between positive and negative slopes occurring at progressively lower migration rates with increasing number of loci (Fig. 4). By contrast, in the case with redundancy, there was an attenuation in the increase in π_w_ found at high migration rates with an increasing number of loci, but little change in the decrease in π_w_ found at low migration rates (Fig. 5) This attenuation effect was driven by similar patterns across all loci, rather than as a result of taking the arithmetic mean across few loci with strong patterns and many loci with weak patterns (Supplementary Fig. 12). For genotypically redundant traits, we found that roughly 50% of the adaptive loci were highly differentiated between patches when migration was low (i.e. allele frequency differences of 95% or greater) (Supplementary Fig. 13), and that there was little difference in the dd-slopes between divergent and uniform selection regimes across low migration high redundancy parameter space (Supplementary Fig. 14).

Finally, the mutation rate at adaptive loci had little effect on any of the qualitative patterns that were seen over very low migration rates (Supplementary Fig. 15). In contrast, across higher migration rates, increasing the mutation rate attenuated both the increase in π_w_ as well as the increase in divergence observed near the selected loci in the parameter sets with a small number of loci (Supplementary Fig. 15). Note that the majority of our simulations were performed at *μ* = 10^−5^ where transient genomic architectures did not play a strong role (Supplementary Fig. 16). Increasing the mutation rate further could increase what influence transience might have on our results.

### Single-locus, ten-patch model

**Fig. 6.**
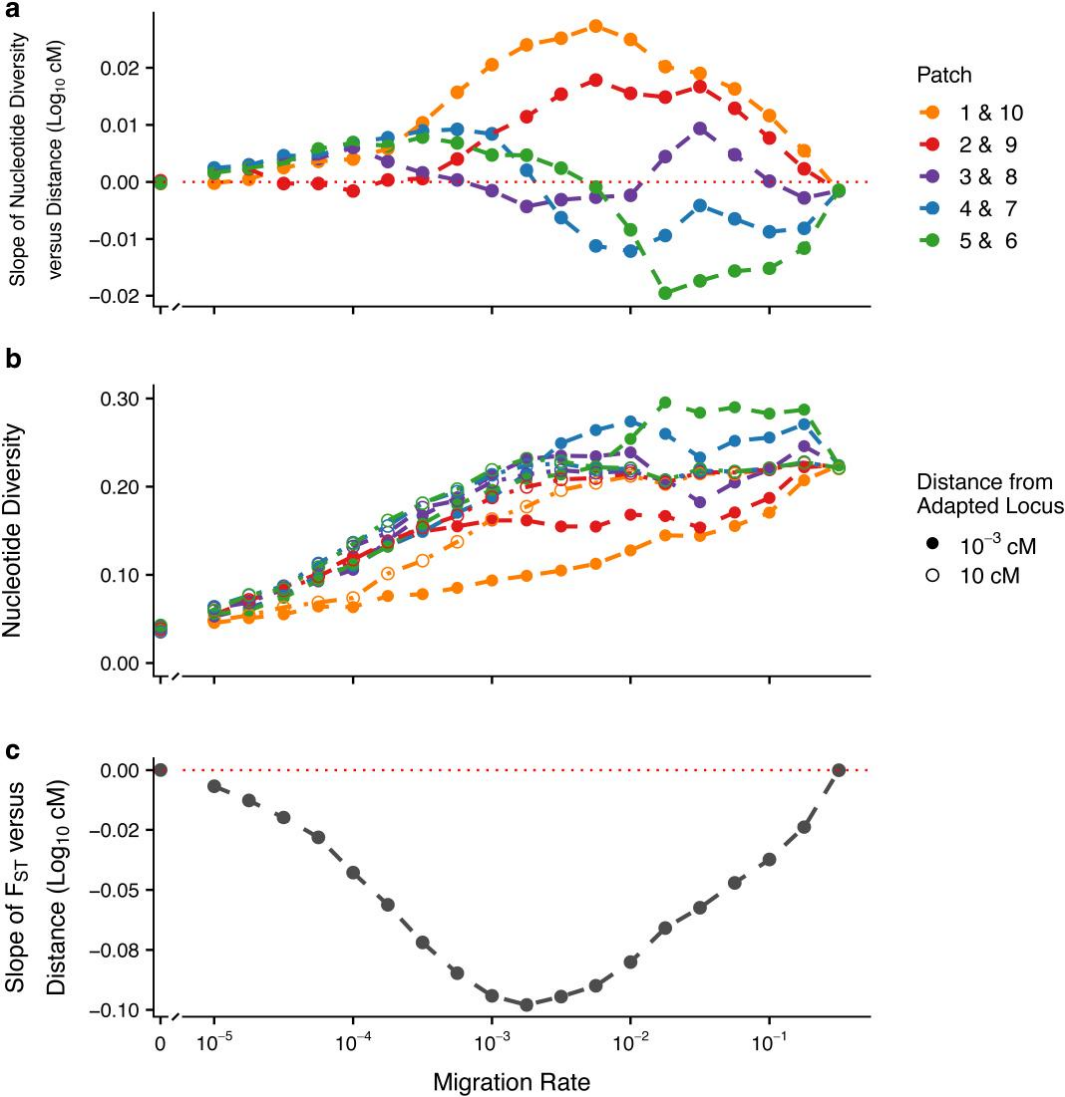
Effect of migration–selection balance on genetic variation at neutral sites linked to a single divergently selected locus in ten-patch model. Each patch was comprised of *N* = 1,000 individuals, mutation rate = 10^−5^ per locus, and *V*_S_ = 5. Panels (a–c) are as described in Fig. 2.

We explored how more realistic models of population structure influenced patterns in π_w_ and F_ST_ by investigating the previously described metrics across a linear, ten-patch environmental gradient. We found that patches on the interior of the landscape (i.e. patches 4–7) produced qualitative patterns very similar in nature to the two-patch model, including positive dd-slopes over low migration rates and negative dd-slopes over intermediate–high migration rates (Fig. 6a-b). Moving towards the exterior of the landscape (i.e. patches 1–3 and 8–10), the dd-slopes were negative over a much reduced region of the migration–selection parameter space explored (Fig. 6a-b). In the patches at either end of the landscape, the dd-slope was positive for all migration rates save for the very extremes (Fig. 6a-b). Similar to the two-patch scenario, the slope of F_ST_ by distance reached a maximum magnitude at an intermediate–high migration rate, before increasing to approximately zero beyond the critical migration rate (Fig. 6c).

In certain regions of migration–selection parameter space (e.g. *m* = 10^−1.75^, *V_S_* = 5) it was possible to find both strong peak and strong trough signatures in a single population, so we further investigated the dynamics at the adaptive locus here to better understand how adaptation over environmental heterogeneity occurs. Adaptation in these regions of parameter space involved the interplay of a number of different adaptive alleles segregating in each patch at once (Supplementary Fig. 17), where patches on the interior of the landscape had a larger number of different alleles relative to those on the periphery (Supplementary Fig. 18). Very generally, populations evolved phenotypic values that approached their local optima through different combinations of two large effect size alleles (∼ ±0.4) and an intermediate allele (∼0): populations on the periphery approached their local optima by being homozygous for a single allele of large effect (i.e. one of ∼ ±0.4), whereas populations in the interior tended to be homozygous for an intermediate allele (∼0) or had two alleles of large effect of opposite signs (Supplementary Fig. 17).

## Discussion

### Local adaptation can cause peaks or troughs in nucleotide diversity

To study how local adaptation in heterogeneous environments shapes patterns in nucleotide diversity within populations, we assessed π_w_ at neutral sites linked to a causal locus driving a trait under spatially divergent selection over a wide range of parameter space. Broadly, we demonstrate that no single signature for π_w_ is characteristic of local adaptation. As most previous work has focused primarily on studying among-population diversity (i.e. F_ST_, d_xy_) as the primary signature of local adaptation, this helps contextualize the contrasting results about π_w_ found in the literature (Petry 1983; Charlesworth *et al*. 1997; Nordborg 1997; Sakamoto and Innan 2019). Given that local adaptation can generate such contrasting patterns in π_w_, we do not advocate for identifying putative genetic signatures of local adaptation solely using patterns in π_w_ at the expense of patterns in among-population diversity, rather, we suggest a more holistic approach, using patterns in π_w_ to help contextualize patterns in among-population diversity. We now summarize how evolutionary processes interact to yield these contrasting patterns in π_w_.

When migration is sufficiently low that migrant haplotypes don’t persist for long and are rapidly selected out of the population, local adaptation produces a pattern resembling background selection (Fig. 2a, positive region, as per Nordborg 1997). In the two-patch model here, this results in π_w_ ∼1.5 × lower than at the unlinked locus (Fig. 2b), which experiences an effective population size more congruent with that of the metapopulation as a whole (Whitlock and Barton 1997). The widths of the regions of depleted π_w_ observed here are similar to the expectations for background selection (as per Nordborg 1997), where diversity is predicted to be depleted on the order of 10's of cM away from the causal locus under similar conditions (Hudson and Kaplan 1995). By contrast, with selective sweeps, diversity is predicted to be depleted more deeply, but over a narrower region, on the order of centiMorgans away from a focal locus (Barton 2000). While the width of the region observed here is similar to the expected width under background selection (Supplementary Fig. 6a), the magnitude of depletion is substantially greater with local adaptation (Fig. 2a). The effect of background selection can be seen when *m* = 0, as both populations evolve towards their respective equilibria and further mutations are deleterious and are selected against.

It is interesting that our simulations show that as migration rate decreases, π_w_ at tightly linked sites tends towards the purely neutral expectation for a single deme without migration (4*N*_e_*μ*), approaching this across higher migration rates than observed for loosely linked sites (Fig. 2b). At first glance, these results can appear to conflict with the classic prediction from a purely neutral island model, where mean π_w_ = 4*dN*_e_*μ*, which is insensitive to migration (Maruyama 1971; Nei and Feldman 1972): if there is no net effect of selection on a weakly linked neutral locus, then should not π_w_ also be insensitive to migration? One factor that differs between these approaches is time: the analytical models provide equilibrium solutions, whereas our simulations report patterns after 25*Nd* generations (see Supplementary Fig. 11 for temporal change). The time to approach equilibrium in such models can exceed millions of generations at low migration rates (Supplementary Fig. 5), so considering both the equilibrium conditions and patterns during approach provides some insight. However, there is also a more fundamental problem to contend with in theoretical studies: is using the arithmetic mean as a summary statistic actually biologically representative? The classic (and surprising) result that π_w_ = 4*dN*_e_*μ* is insensitive to migration occurs because the arithmetic mean is taken across two types of evolutionary behavior: replicates that have recently undergone a migration event (and have elevated π_w_), and those that have not (Nordborg 1997; Wakeley 2009). As migration rate decreases, F_ST_ increases and each migrant causes a greater inflation in π_w_, but the proportion of replicates with a recent migrant tends towards zero, becoming essentially undetectable by simulations studies that are computationally constrained to a finite number of replicates (e.g. Supplementary Fig. 5). Thus, in simulations with a modest number of replicates, the arithmetic mean across replicates does not yield the classic analytical result, and is instead “biased” towards the expectation for replicates without a recent migrant (which tend to be the only ones present when a modest number of replicates have been run).

But is the classic analytical result of π_w_ = 4*dN*_e_*μ* actually representative of biology? In simulations of the purely neutral model at low migration rates (Supplementary Fig. 5) almost all individual replicates have π_w_ either much higher than 4*dN*_e_*μ*, or much closer to 4*N*_e_*μ*, and almost no replicate has π_w_ = 4*dN*_e_*μ*. Similarly, while our simulation results with selection and linkage may fail to capture the impact of the rare replicate with a rare recent migrant on observed mean π_w_ at low migration rates, the average observed π_w_ across simulation replicates more realistically captures the expectation for a typical metapopulation (indeed, a more accurate way to portray results would be to show the distribution of π_w_ across replicates). The decrease in π_w_ at tightly linked sites that we observe at low migration rates and strong selection can therefore be interpreted as the most likely evolutionary outcome, such that π_w_ tends towards the expectation for a single deme. Of course, appropriate considerations must also be made for the temporal change in such patterns as populations approach equilibrium, or are perturbed away from it. Given the myriad ways that different evolutionary scenarios can generate peaks or troughs in diversity, and the considerable heterogeneity observed around the expectation for a given parameter set (Supplementary Fig. 5), it is difficult to make any clear inference for any given point observation of a peak or trough in empirical data.

Based on these results, we would predict that particularly extreme reductions in π_w_ at linked sites might be seen in small peripheral populations experiencing weak migration and strong selection. In this case, neutral regions of the genome would have levels of π_w_ similar to those expected for the effective size of the metapopulation, whereas loci linked to the selected locus would have levels of π_w_ similar to those expected for the effective size of the small peripheral population, which could be much more discordant than found with the symmetrical population sizes simulated here. In such regions of parameter space where we find an erosion of π_w_ at the neutral loci flanking locally adapted loci, we also find an increase in F_ST_ at the same sites (Fig. 2c). Thus, this pattern which has been interpreted as a result of background selection or uniform positive selection (Noor and Bennett 2009; Cruickshank and Hahn 2014), can also be driven by migration–selection balance, as could be predicted from previous migration–selection studies (e.g. Petry 1983; Bengtsson 1985; Barton and Bengtsson 1986; Nordborg 1997). Our results do not discount the effects positive selection or purifying selection may have on producing signatures resembling the classic “genomic islands of differentiation” (e.g. Nosil *et al*. 2009; Via 2009), but do show that local adaptation could also generate similar patterns in nucleotide diversity and F_ST_.

Conversely, when migration is higher but not so strong as to collapse the locally adapted polymorphism, we find peaks in both π_w_ (Fig. 2a, negative region) and F_ST_ (Fig. 2c) at the neutral loci flanking locally adapted loci, although this effect is attenuated with larger population size (Fig. 3). Selection generates linkage disequilibrium between neutral loci and the locally adapted locus proportional to the recombination distance between them; when the locally adapted haplotype migrates into its maladapted patch, π_w_ is transiently increased at the flanking neutral loci. When locally adapted haplotypes migrate into the maladapted patch at a greater rate than selection can effectively purge them, sharp peaks are generated in both π_w_ and F_ST_. These peaks attenuate with larger population sizes, as the maintenance of linkage disequilibrium is increased over a greater range of recombination with smaller populations (as per Ohta and Kimura 1971). While a limited effect of increased diversity was noted by Sakamoto and Innan (2019), this was not discussed as a potentially important signature of local adaptation. Here, we show that considerable increases in π_w_ can be found, especially when selection is strong, migration rate high, and effective population sizes are small, as is the case in many empirical examples of local adaptation. Concurrent peaks in π_w_ and F_ST_ may therefore constitute an important signature of local adaptation that can be readily distinguished from background and positive selection—not all local adaptation will cause increased π_w_ along with peaks in F_ST_, but the detection of such patterns strongly suggests local adaptation as a driving process.

It is perhaps interesting to contrast these results with the expectation for standing genetic variation (*V*_G_) in a quantitative trait, which shows an increase in *V*_G_ under high migration rates, but no reduction in *V*_G_ under low migration rates (McDonald and Yeaman 2018). This further highlights the importance of clearly specifying model expectations, and the problems inherent in using nucleotide diversity data as a proxy for *V*_G_ (as per Reed and Franhkam 2001). Clearly, the question of “what maintains variation?” has a very different meaning for different kinds of variation.

### Effects of genotypic redundancy

For traits that have no genotypic redundancy, the effect of selection on the phenotype is divided among loci in proportion to their effect sizes (Yeaman 2015). As such, the strength of selection per locus is reduced as the number of loci increases, and the peaks and troughs in π_w_ at linked sites also attenuate, as described above (Fig. 4). In contrast, when traits are genotypically redundant, we still find strong trough-like patterns in π_w_ as the number of loci increases (Fig. 5). When migration is low (*m* ≪ 1/*N*_e_), different populations evolve essentially as though they were independent of one another, with each population converging on a certain combination of alleles that achieves its phenotypic optimum effectively independent of the particular combination of alleles the other population is using. As a result, the two different populations tend to evolve very different combinations of alleles and end up differentiated at substantially more loci than would strictly be needed to achieve local adaptation in each patch (Supplementary Fig. 13). When the different populations are differentiated at such a large proportion of loci, matings between divergently adapted individuals result in F2 hybrid breakdown (Yeaman and Whitlock 2011; Thompson *et al*. 2019), causing selection to operate on many more loci than would be expected solely due to local adaptation. The action of selection on all differentiated loci thereby causes the background selection-like effect across many loci, and prevents the attenuation of the trough-like pattern at low migration rates and many redundant loci. Furthermore, the population structure that is generated through low migration and high genotypic redundancy (as per Goldstein and Holsinger 1992; Phillips 1996) generates a similar trough pattern even with spatially uniform selection (Supplementary Fig. 14). If many genotypically redundant loci scattered throughout the genome contribute to local adaptation, this could cause an extensive decrease in π_w_ at low migration rates. Studying the prevalence of F2 hybrid breakdown among divergent populations or sub-species could help indicate whether highly redundant traits causing this kind of effect are common.

### Peaks and troughs in diversity under more realistic models of population structure

When a species range spans an environmental gradient, populations that inhabit the interior regions experience very different evolutionary processes from those inhabiting the periphery, due to variations in migration rate combined with the different combinations of allele frequencies found in different regions. We can see the effects of variable evolutionary processes over the species range in our ten-patch model, where patterns in π_w_ are starkly different between the populations that inhabit the interior of the range and those that inhabit the periphery (Fig. 6). Populations that inhabit the interior of the range have patterns in π_w_ that are qualitatively quite similar to those described in our two-patch model; in contrast, populations that inhabit the periphery present with depleted π_w_ across a substantially larger region of migration–selection parameter space and the sharp peaks in π_w_ seen in our two-patch results do not appear (Fig. 6).

Across a spatially extended environmental gradient, the interior populations receive an influx of maladapted alleles at a relatively high rate (here, ≳2 × compared to the peripheral populations), which increases the π_w_ at flanking loci in these populations. While migration is introducing differently adapted alleles into the interior populations, however, selection is purging them. The purging of maladapted alleles across the interior populations, coupled with the stepping stone nature of our model, ultimately results in the decreased influx of maladapted alleles into the peripheral populations (i.e. few haplotypes with an allele optimally adapted to one end of the landscape migrate to the other end). As such, the degree of polymorphism that is maintained around the locally adapted locus in the peripheral populations is not sufficient enough to produce the peaks in π_w_ seen in our two-patch results. Consequently, when species adapt over spatially extended environmental gradients, it may be very possible to find both peaks and troughs in π_w_ at a single-locus within a single species.

### Conclusion

We demonstrate that there is no universal nucleotide-scale signature of local adaptation, even with the simplest possible model of spatially divergent selection. Nucleotide diversity within populations can be substantially decreased or increased depending on the relative strengths of migration and selection. Additionally, local adaptation can result in regions of depleted within-population diversity over chromosomal distances similar to that of background selection, with a substantially greater magnitude of diversity eroded than with background selection. Our results demonstrate that local adaptation must also be considered, in addition to background selection and selective sweeps, when making inferences based on genomic regions of reduced within-population diversity. While reductions in diversity may not be particularly diagnostic, peaks in within-nucleotide diversity are only expected under local adaptation or other models of balancing selection, and as such, can distinguish local adaptation vs uniform positive/purifying selection (as per Booker *et al*. 2021). Finally, our results from models with increased realism further highlight that there is little reason to expect a consistent pattern in within-population nucleotide diversity across heterogeneous environments, as patterns of decreased or increased diversity can be expected depending upon polygenicity, redundancy, geography, migration, and selection.

## Supplementary Material

jkae225_Supplementary_Data

## Data Availability

Nemo initialization files and code to calculate and plot diversity metrics are available on github.com/russjasp/peaks_and_troughs. The authors affirm that all data necessary for confirming the conclusions of the article are present within the article, figures, and table. Supplemental material available at G3 online.
