# Supplementary material for: Local adaptation can cause both peaks and troughs in nucleotide diversity within populations
Source: G3 (Bethesda). 2024 Sep 18;14(11):jkae225. doi: 10.1093/g3journal/jkae225 (PMC11540321; doi:10.1093/g3journal/jkae225)
Supplement: jkae225_Supplementary_Data [file jkae225_supplementary_data.pdf]

SUPPLEMENTAL INFORMATION

Supplemental Methods

To illustrate the behaviour of simulations a neutral model with a limited number of replicates (Fig. S4), we ran additional sets of simulations in both SLiM3 (Haller and Messer 2019) and ms (Hudson 2002). SLiM3 was run using standard Wright-Fisher default settings, with recombination rate of 10^{-8} between adjacent base pairs and full output of simulated individual genotypes. Results were processed in R, and nucleotide diversity was calculated as the average expected heterozygosity of all variant sites divided by the number of base pairs (10^5). Coalescent simulations with ms were run using default settings, with all parameters indicated in the main text. Coalescent migration rates were converted to individual-migration rates prior to plotting.

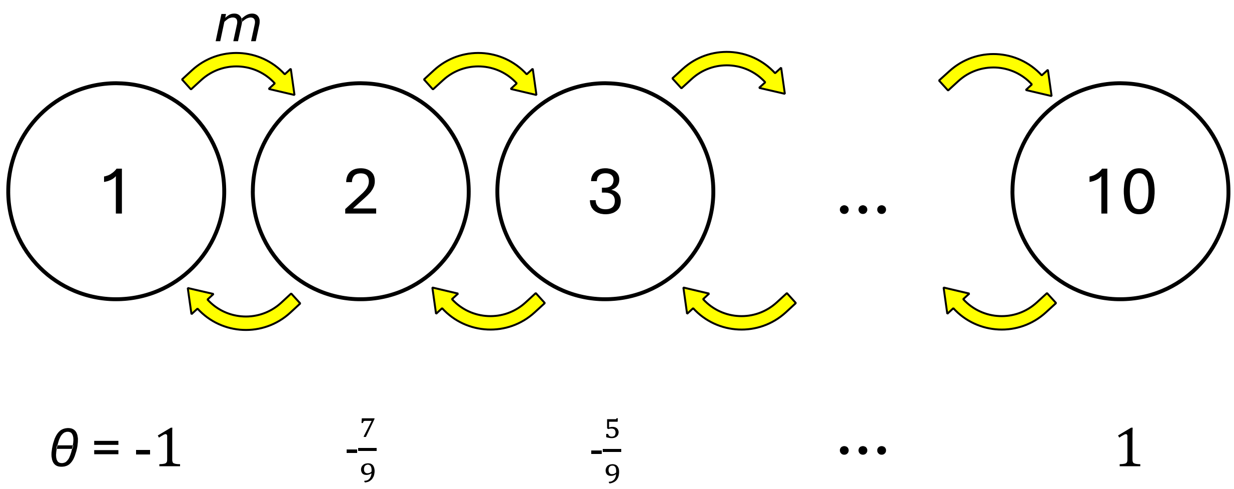

Figure S1: Schematic of ten-patch model. Patches were arranged linearly and followed the stepping stone model of migration at rate m. The phenotypic optimum (θ) scaled linearly across the patches.

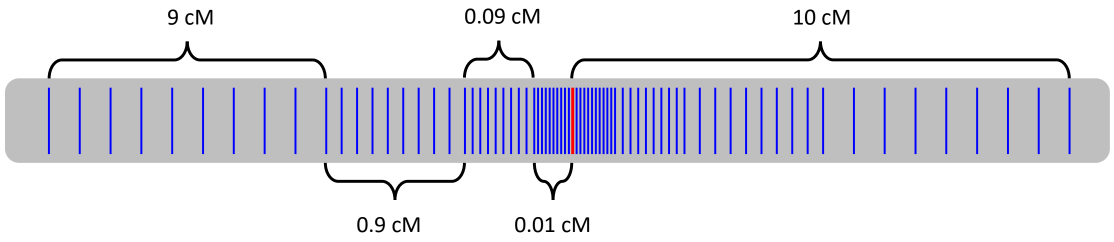

Figure S2: Genetic map of chromosome with a single divergently selected locus. The chromosome consisted of one divergently selected locus and 74 neutral loci. The selected locus is denoted by the red bar, the neutral loci are denoted by the blue bars. The neutral loci were positioned about the selected locus symmetrically at distances from 10^{-3} to 10^{1} cM away on a $\log_{10}$ scale.

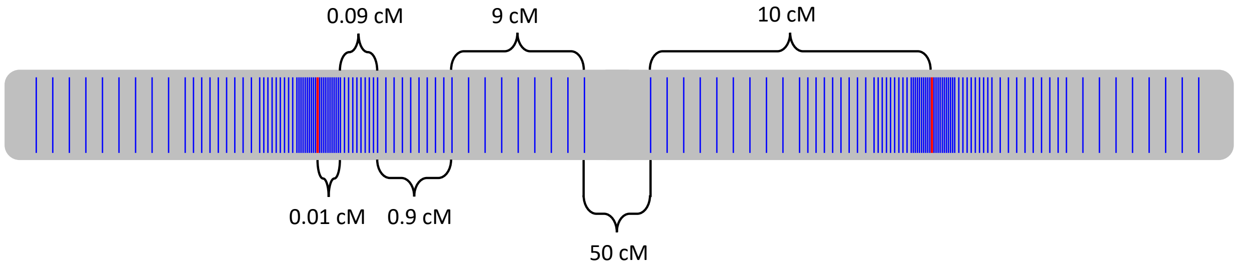

Figure S3: Genetic map of chromosome with multiple divergently selected loci. Each complement of a selected locus and its 74 flanking neutral loci was separated by at least 50 cM. All genetic architectures with more than a single selected locus followed this same pattern.

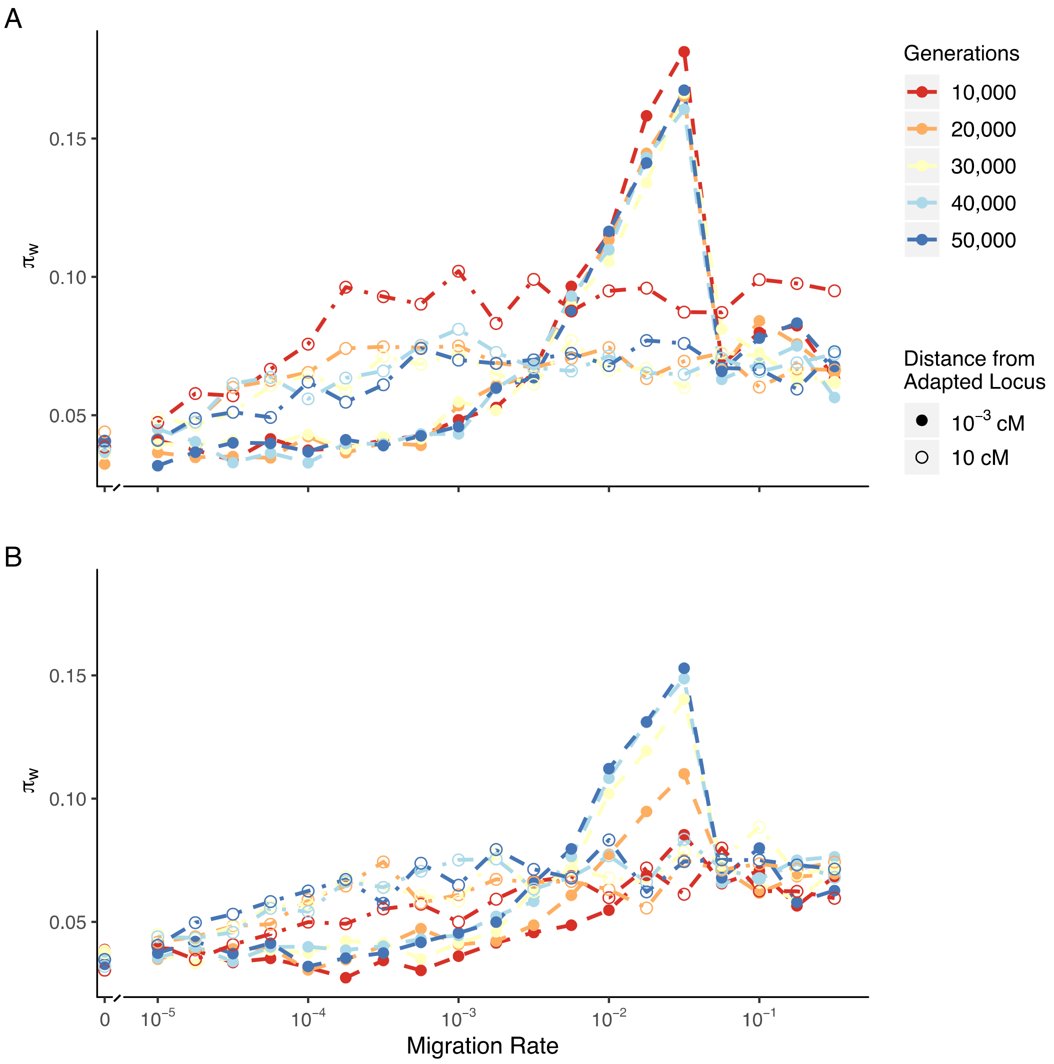

Figure S4: Time series effect of neutral allele initialization model and migration-selection balance on π_w at neutral sites linked to a single divergently selected locus in two-patch model. The π_w at the loci closest (10^{-3} cM) and furthest (10 cM) from the locally adapted locus is shown using a neutral allele initialization model whereby loci are randomly prescribed an allele value (A) and whereby all loci are initialized with the same allele value (B). Each patch was comprised of N = 1,000 individuals, mutation rate per locus = 10^{-5}, and V_S = 5.

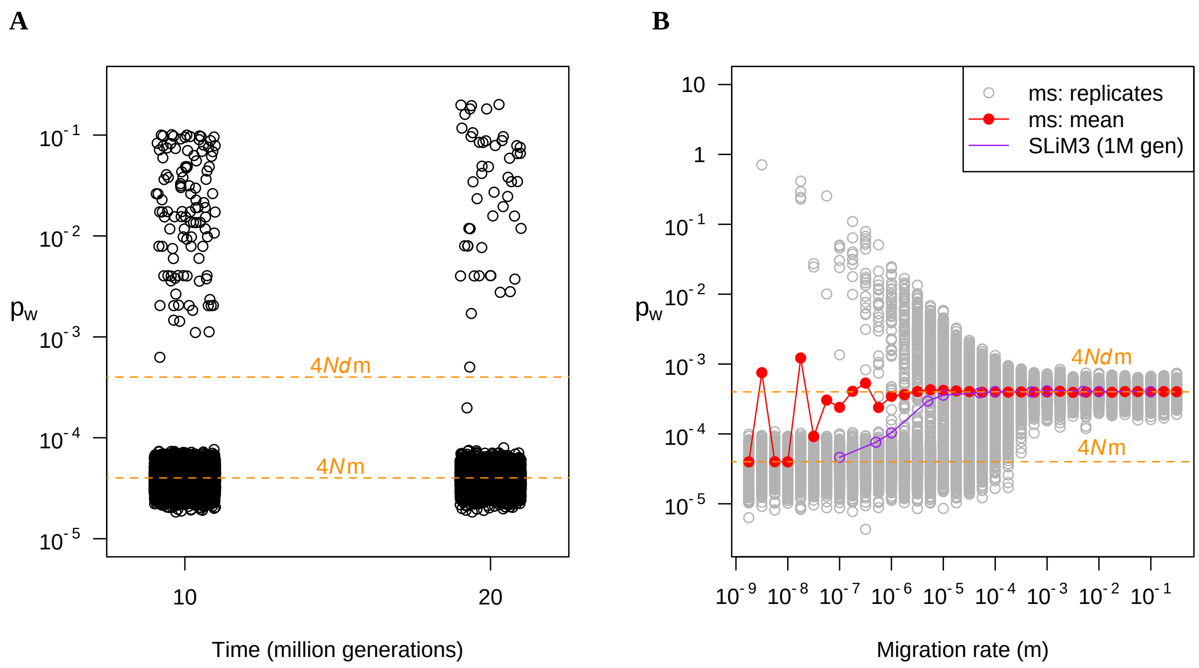

Figure S5. Nucleotide diversity within populations (π_W) has a bimodal distribution at lower migration rates. Panel A shows results for forwards-in-time simulations run using SLiM3 with $m = 10^{-7}$, sampled after 10 and 20 million generations of evolution, while panel B shows results for coalescent simulations in ms across a wider range of migration rates, along with SLiM3 results sampled after 10^6 generations. In all cases, simulations follow a 10-deme island model with $\theta = 0.4$ and track a 100 kbp sequence. In panel A, SLiM3 simulations have $N = 100$ and $\mu = 10^{-7}$. In panel B, SLiM3 simulations have $N = 1{,}000$, $\mu = 10^{-8}$.

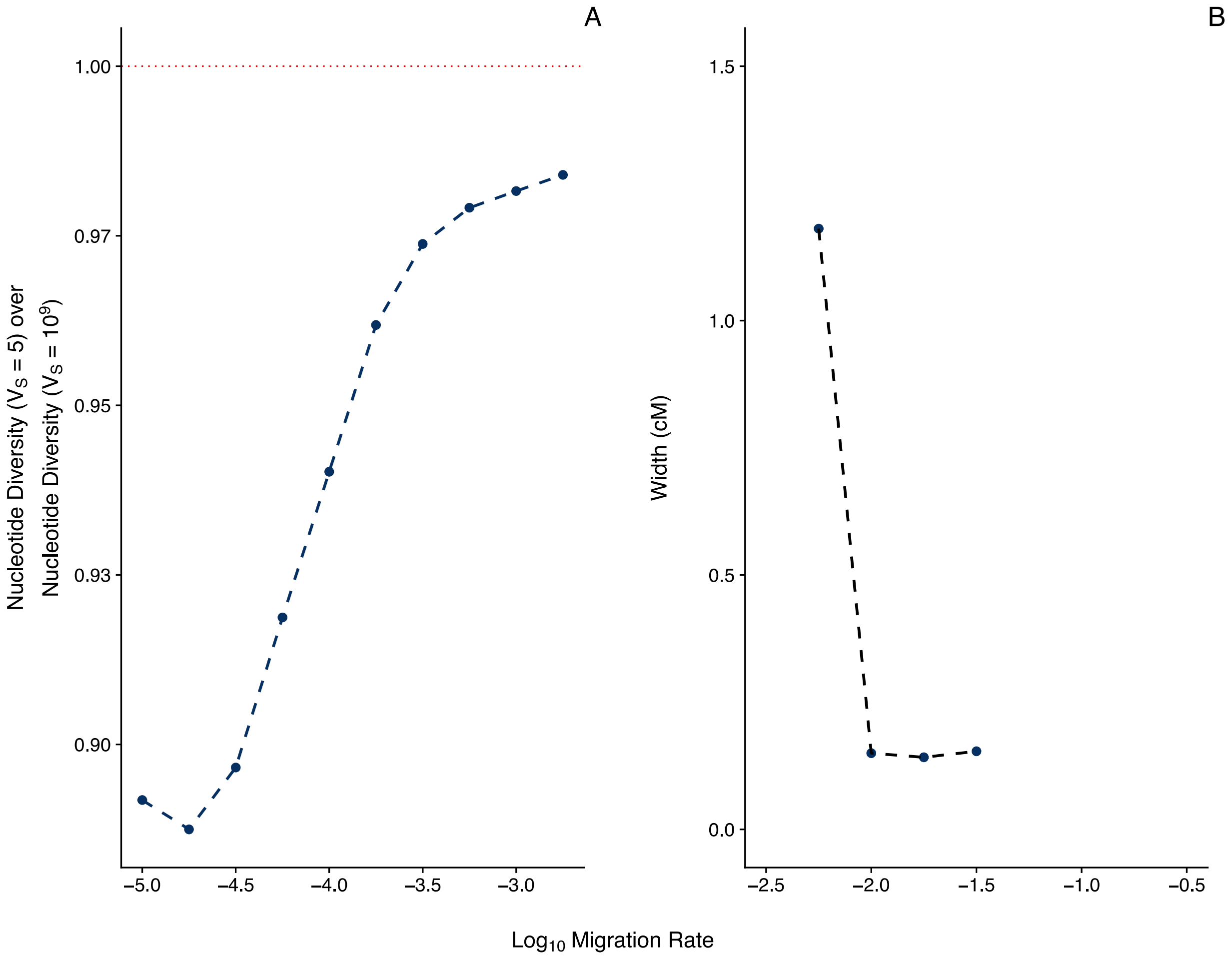

Figure S6: Genetic distance over which diversity at neutral sites is affected by linkage to a single divergently selected locus in two-patch model. Panel A shows the degree to which within-population nucleotide diversity is eroded at the neutral loci 9 to 10 cM away from the locally adapted locus under strong selection (V_S = 5) relative to genome-wide background levels ($V_S = 10^9$). Panel B shows the width (cM) of peaks in within-population nucleotide diversity at the neutral loci linked to the locally adapted locus. Each patch was comprised of N = 1,000 individuals and the mutation rate = 10^{-5} per locus.

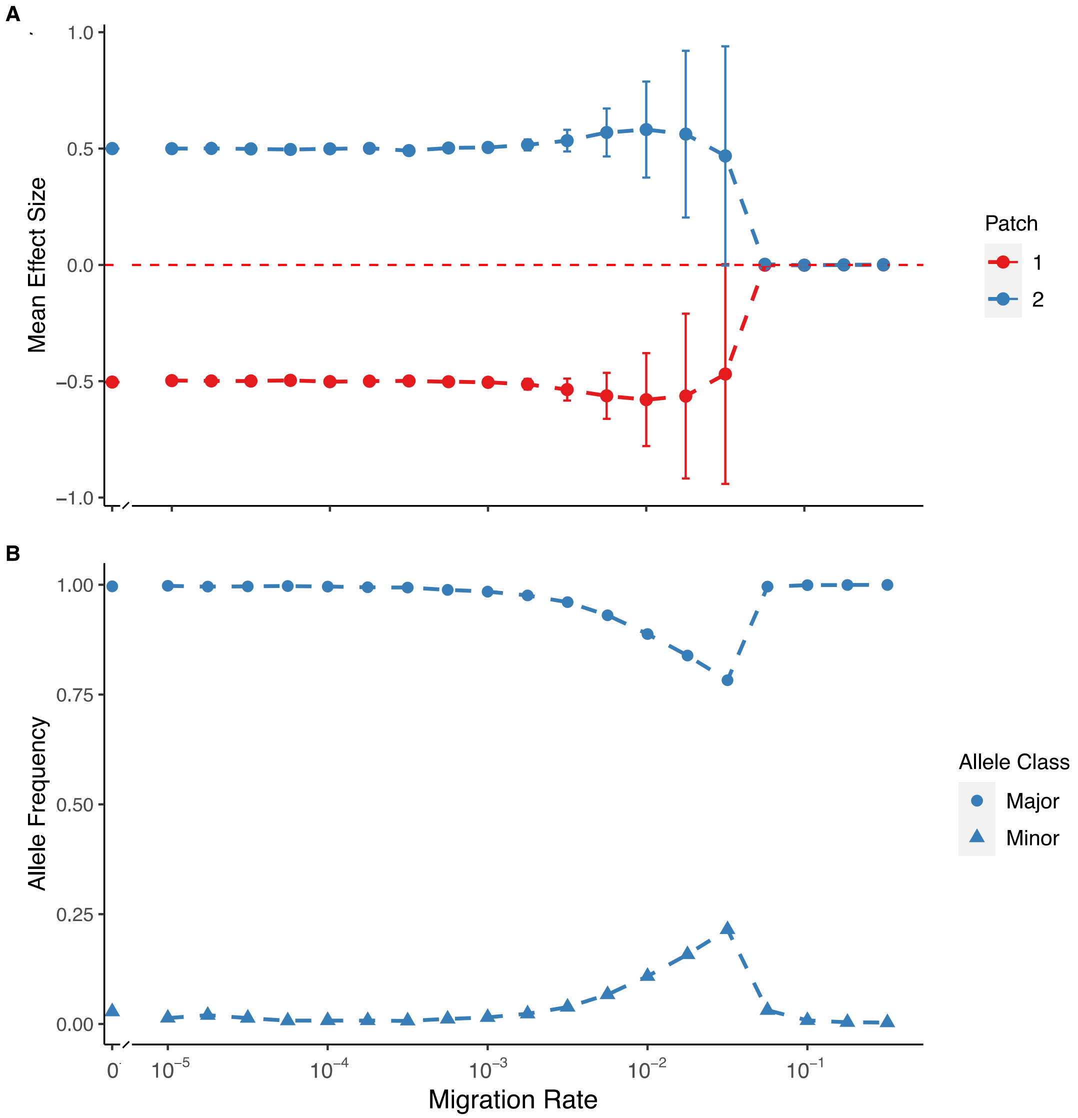

Figure S7: Mean allele effect size and frequency at a single divergently selected locus in two-patch model. The allele effect size mean and variance are shown for each patch against the $\log_{10}$ migration rate (A). Phenotypic optima were -1 and +1 for patches 1 and 2, respectively. The allele frequencies of the major and minor alleles are shown for a given patch (B). Results after 50,000 generations shown; each patch was comprised of N = 1,000 individuals, mutation rate = 10^{-5} per locus, and V_S = 5.

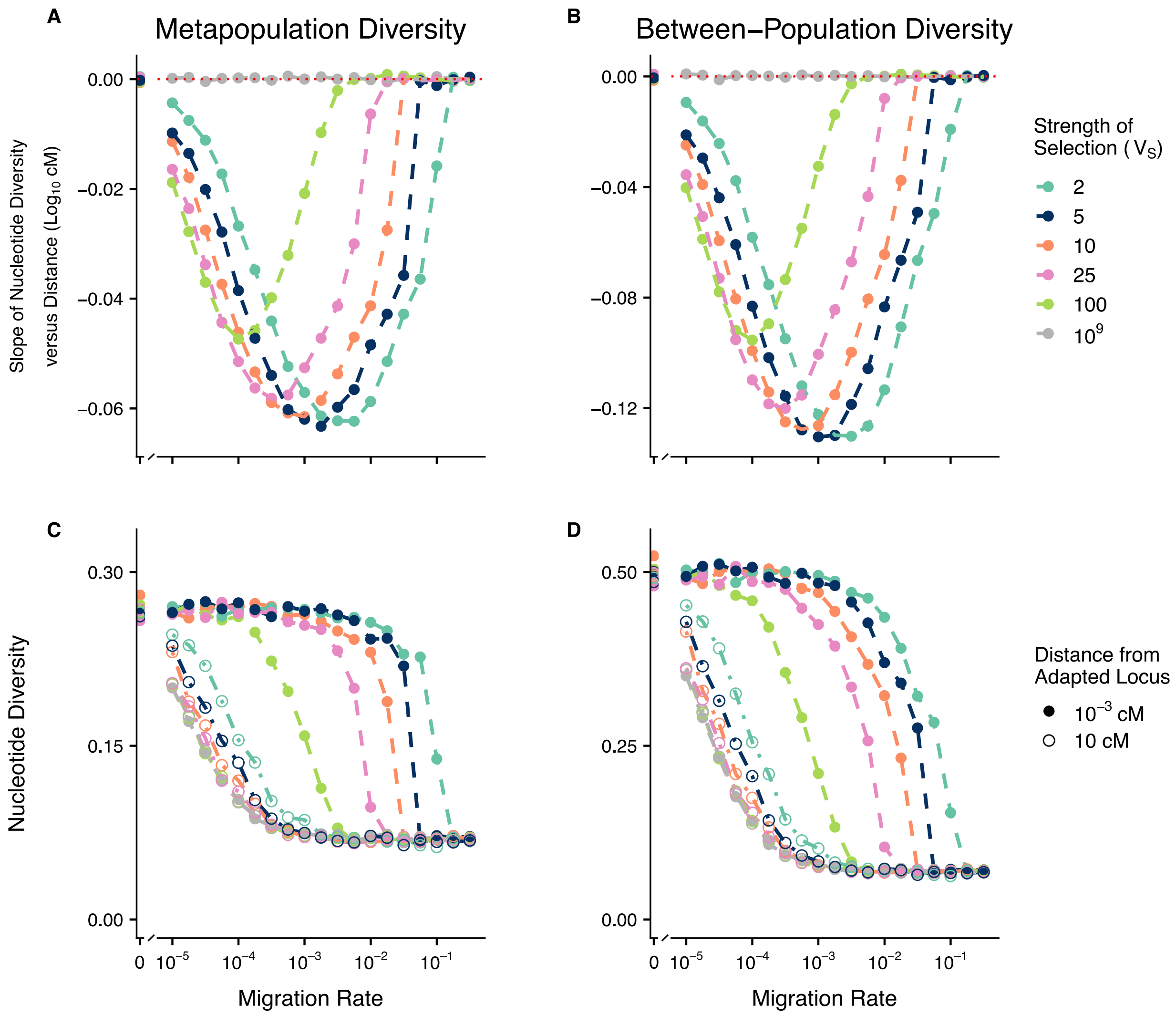

Figure S8: Effect of migration-selection balance on total metapopulation diversity and between-population diversity (d_{xy}) at neutral sites linked to a single divergently selected locus in two-patch model. The slopes of metapopulation nucleotide diversity versus distance ($\log_{10}$ cM) (A) and between-population nucleotide diversity versus distance ($\log_{10}$ cM) (B), and the metapopulation nucleotide diversity (C) and between-population nucleotide diversity (D) at the loci closest (10^{-3} cM) and furthest (10 cM) from the locally adapted locus are shown against the $\log_{10}$ migration rate after 50,000 generations. Each patch was comprised of N = 1,000 individuals and the per-locus mutation rate = 10^{-5}.

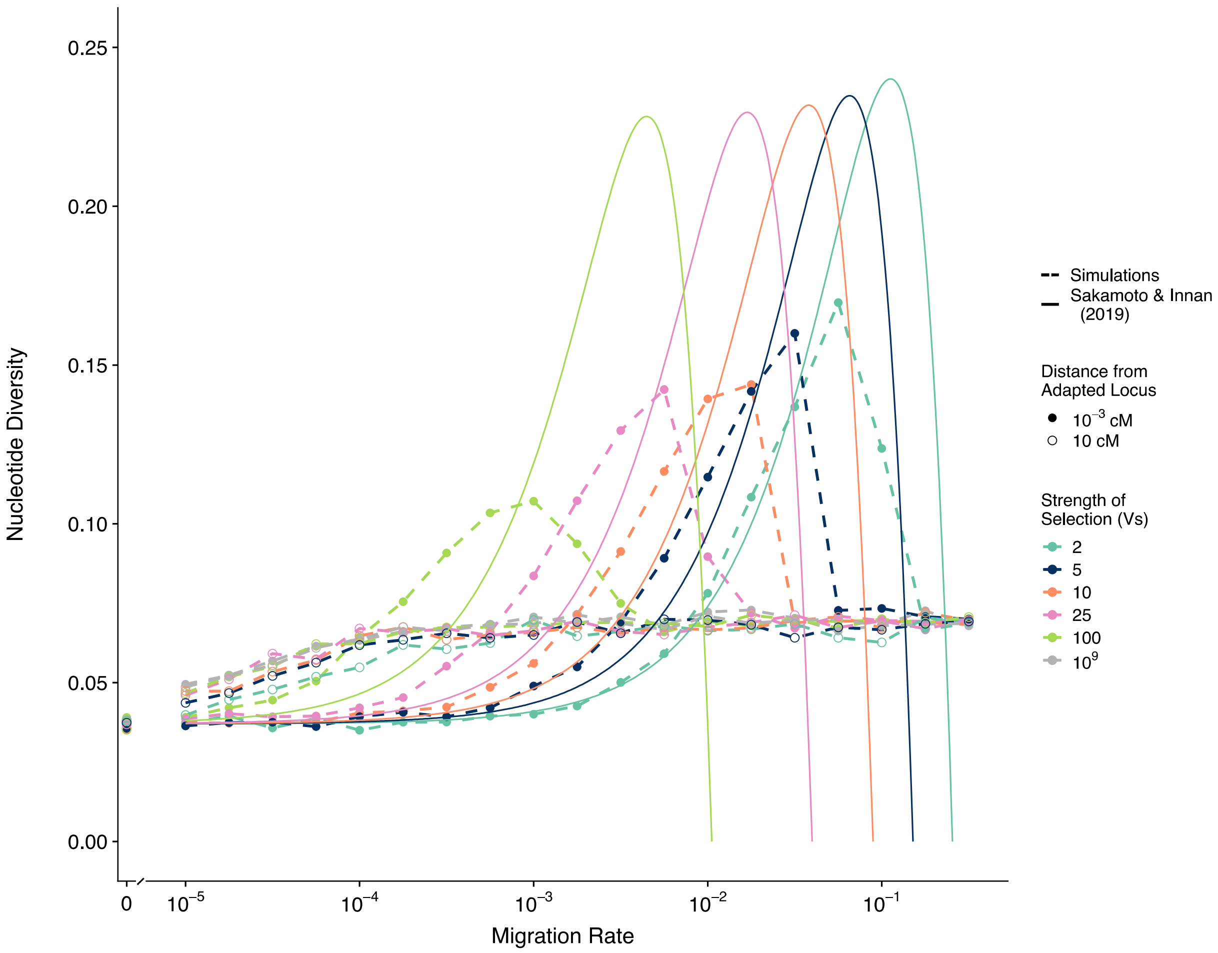

Figure S9: Comparison between simulations results and analytical predictions at a neutral site linked to a single divergently selected locus in two-patch model. Simulation results for the within-population nucleotide diversity at a neutral locus 10^{-3} cM from a divergently selected locus are shown against the $\log_{10}$ migration rate after 50,000 generations. Each patch was comprised of N = 1,000 individuals and the per-locus mutation rate = 10^{-5}. Analytical predictions for the expected within-population heterozygosity at a similarly linked neutral locus from Sakamoto and Innan (2019) equation 25 are shown for comparison. Analytical predictions were generated using symmetrical selection coefficients to parameterize eq. 25 (i.e. their $s_1 = |s_2|$). Simulation results are shown in dashed lines, analytical predictions are shown in solid lines.

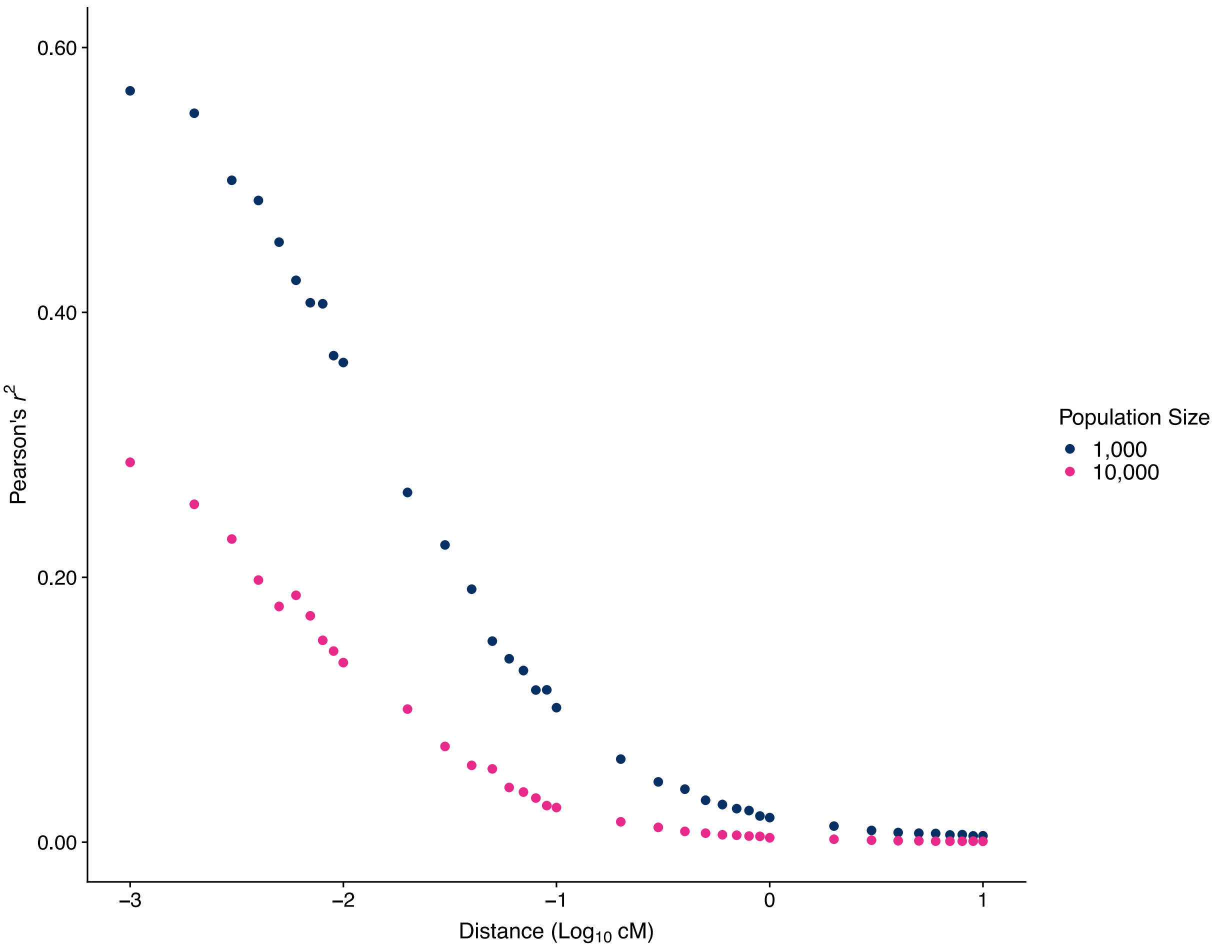

Figure S10: Effect of population size on linkage disequilibrium between neutral loci and a single divergently selected locus in two-patch model. A migration rate of $10^{-1.5}$, mutation rate of 10^{-5} per locus, and V_S of 5 is shown.

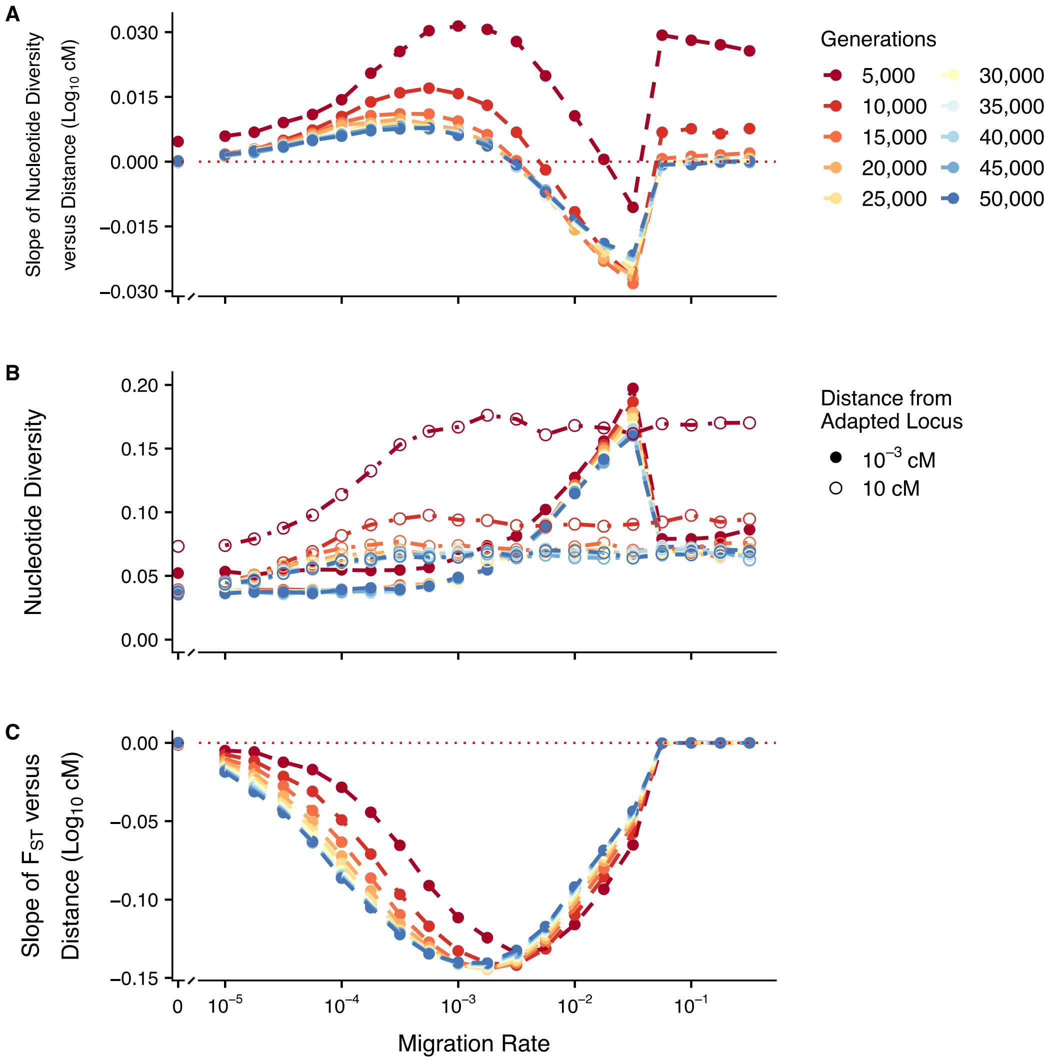

Figure S11: Time series effect of migration-selection balance on diversity at neutral sites linked to a single divergently selected locus in two-patch model. Each patch was comprised of N = 1,000 individuals, mutation rate per locus = 10^{-5}, and V_S = 5. Panels are as described in Fig. 2 in 5,000 generation intervals.

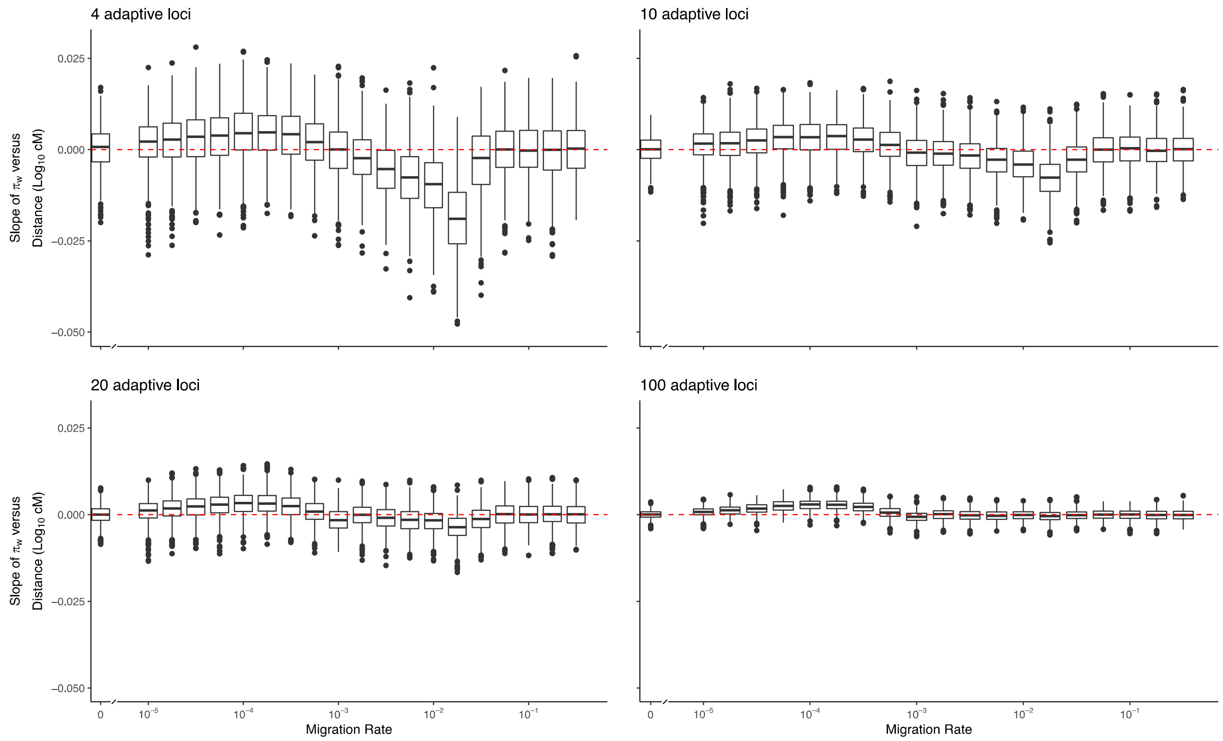

Figure S12: Effect of migration-selection balance on genetic variation at linked neutral sites for a quantitative trait with different numbers of loci and variable levels of genotypic redundancy. The distribution of the slopes of π_w versus distance (log_{10} cM) is shown for four levels of genotypic redundancy. Allele effect sizes were ± 0.25, such that an individual could reach the optimum in a patch (± 1) by being homozygous for the optimal allele at 2 loci. Each patch was comprised of N = 1,000 individuals, mutation rate = 10^{-5} per locus, and V_s = 5.

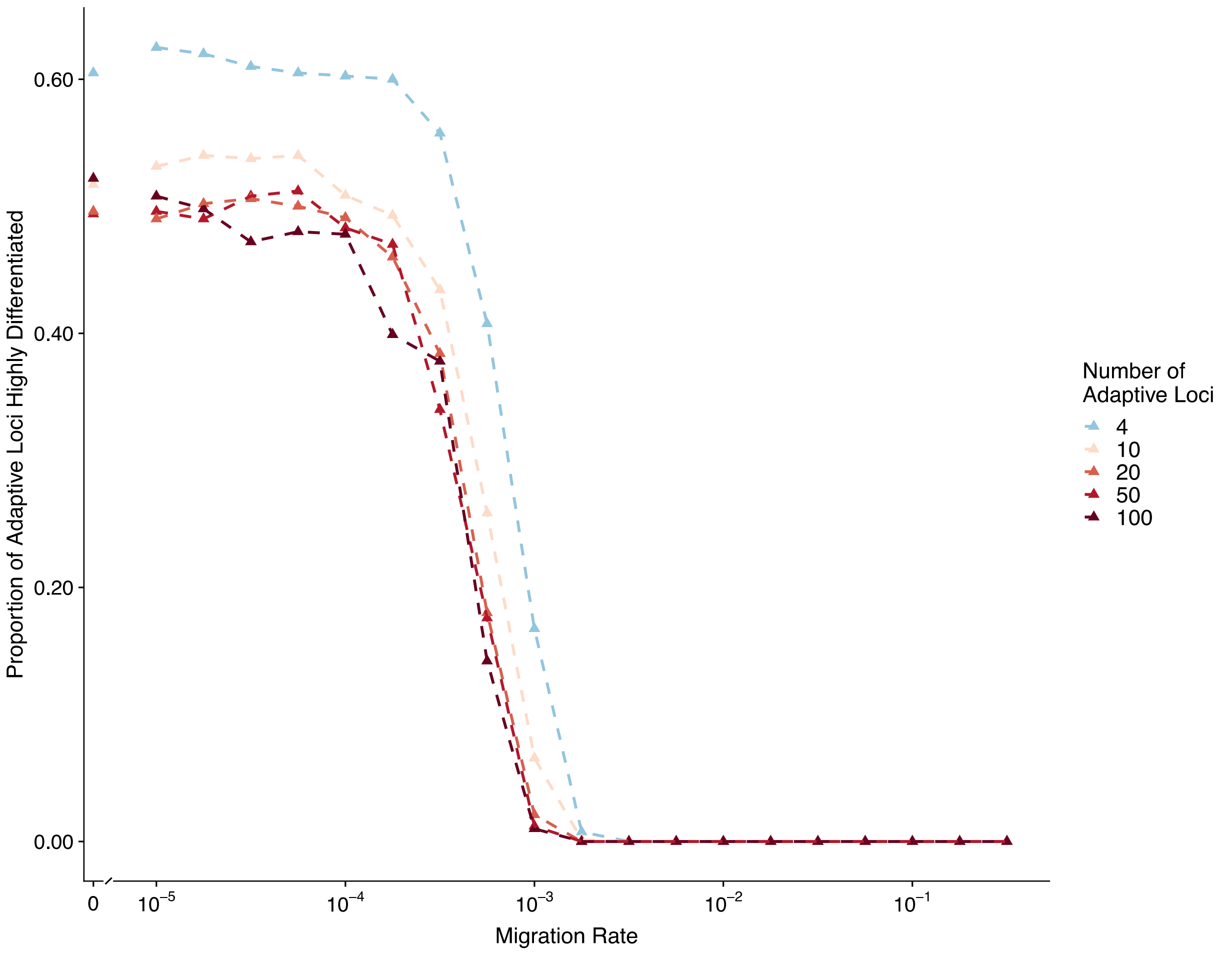

Figure S13: Proportion of adaptive loci highly differentiated between patches for a quantitative trait with different numbers of loci and variable levels of genotypic redundancy. Loci with allele frequency differences of 95% or greater between patches were deemed highly differentiated. Allele effect sizes were ± 0.25, such that an individual could reach the optimum in a patch (± 1) by being homozygous for the optimal allele at 2 loci. Results after 50,000 generations shown; each patch was comprised of N = 1,000 individuals, mutation rate = 10^{-5} per locus, and $V_S = 5$.

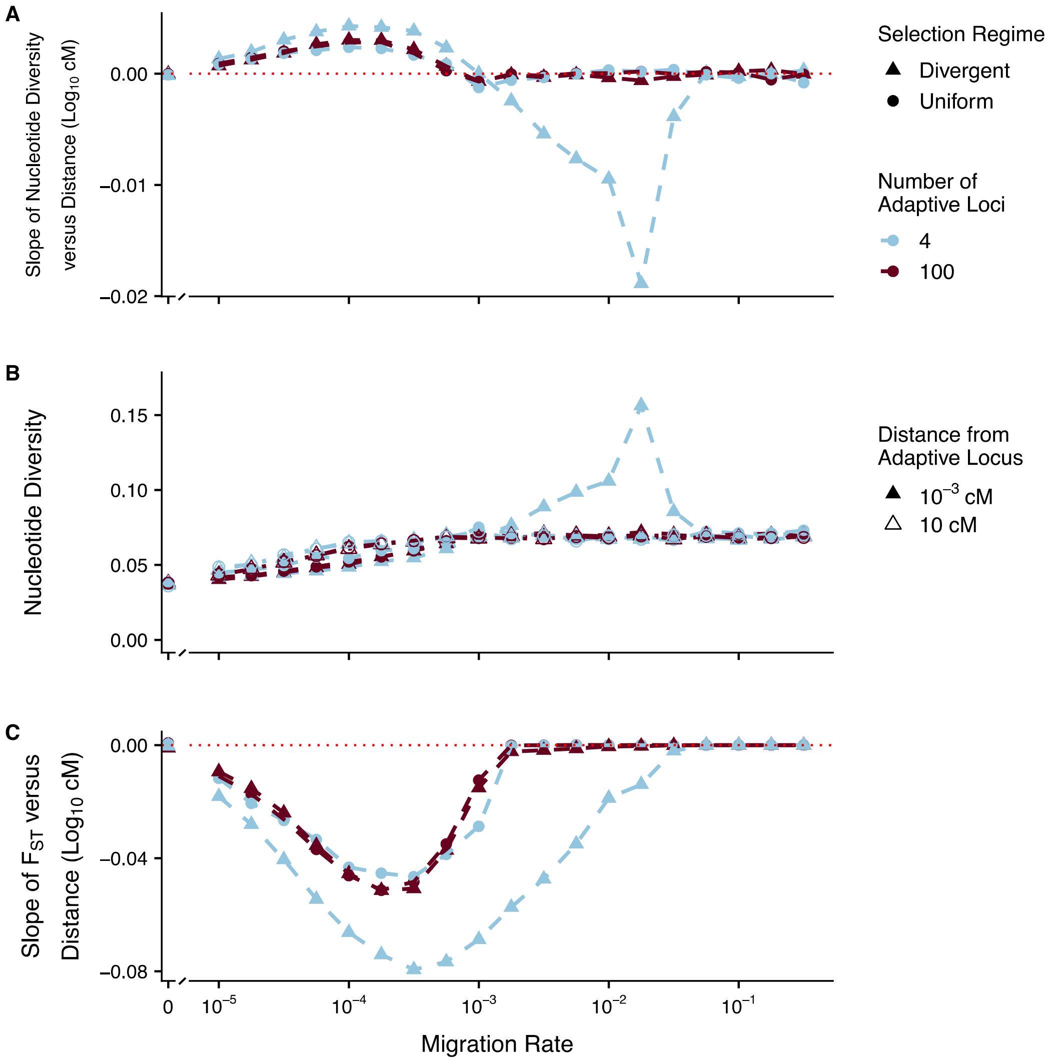

Figure S14: Effect of migration-selection balance and selection regime on diversity at linked neutral sites for a quantitative trait with different numbers of loci and variable levels of genotypic redundancy. Allele effect sizes were ± 0.25, such that an individual could reach the optimum in a patch (± 1 divergent selection; +1 uniform selection) by being homozygous for the optimal allele at 2 loci. Panels are as described in Fig. 2; each patch was comprised of N = 1,000 individuals, mutation rate = 10^{-5} per locus, and V_S = 5.

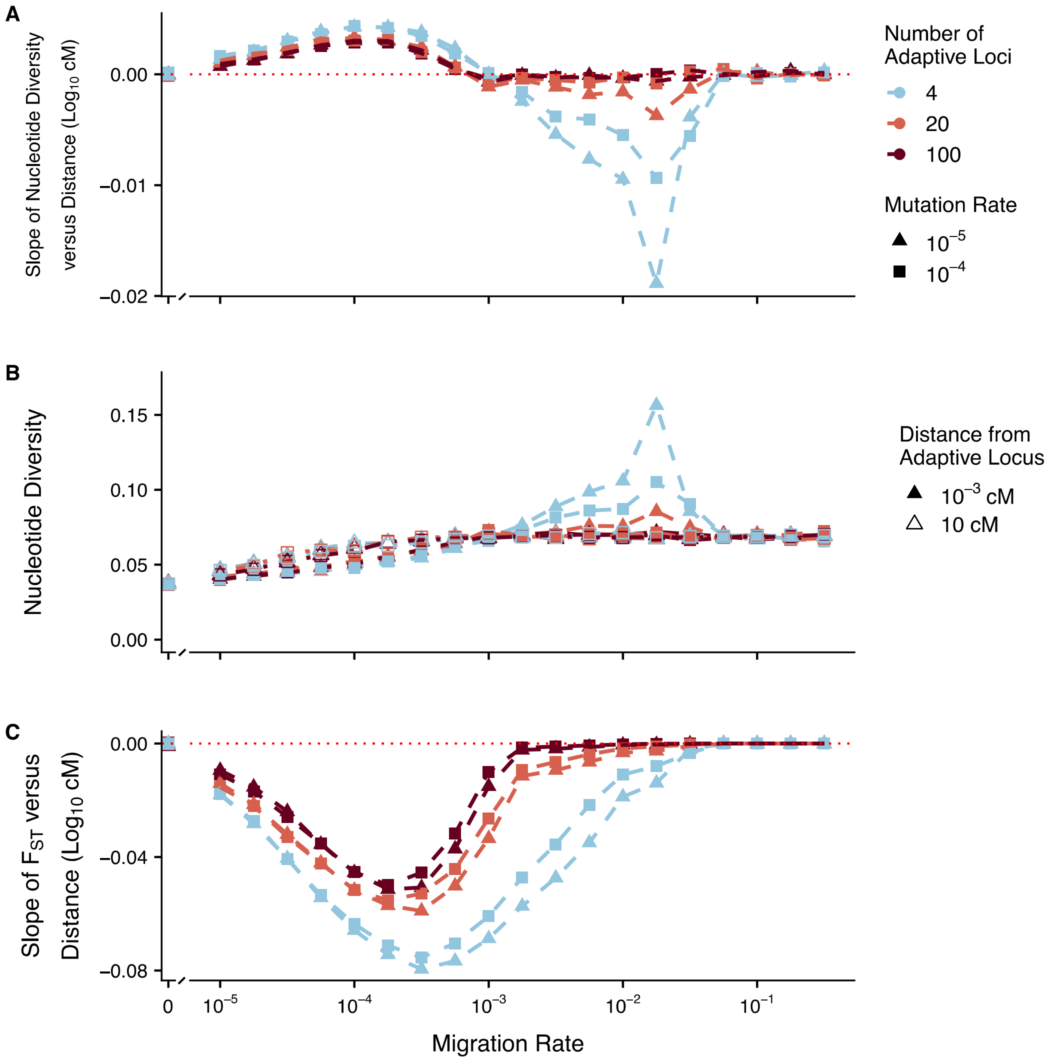

Figure S15: Effect of migration-selection balance and adaptive mutation rate on diversity at linked neutral sites for a quantitative trait with different numbers of loci and variable levels of genotypic redundancy. Allele effect sizes were ± 0.25, such that an individual could reach the optimum in a patch (± 1) by being homozygous for the optimal allele at 2 loci. Mutation rate at selected loci = 10^{-5} or 10^{-4}, and at neutral loci = 10^{-5}. Each patch was comprised of N = 1,000 individuals and V_S = 5. Panels are as described in Fig. 2.

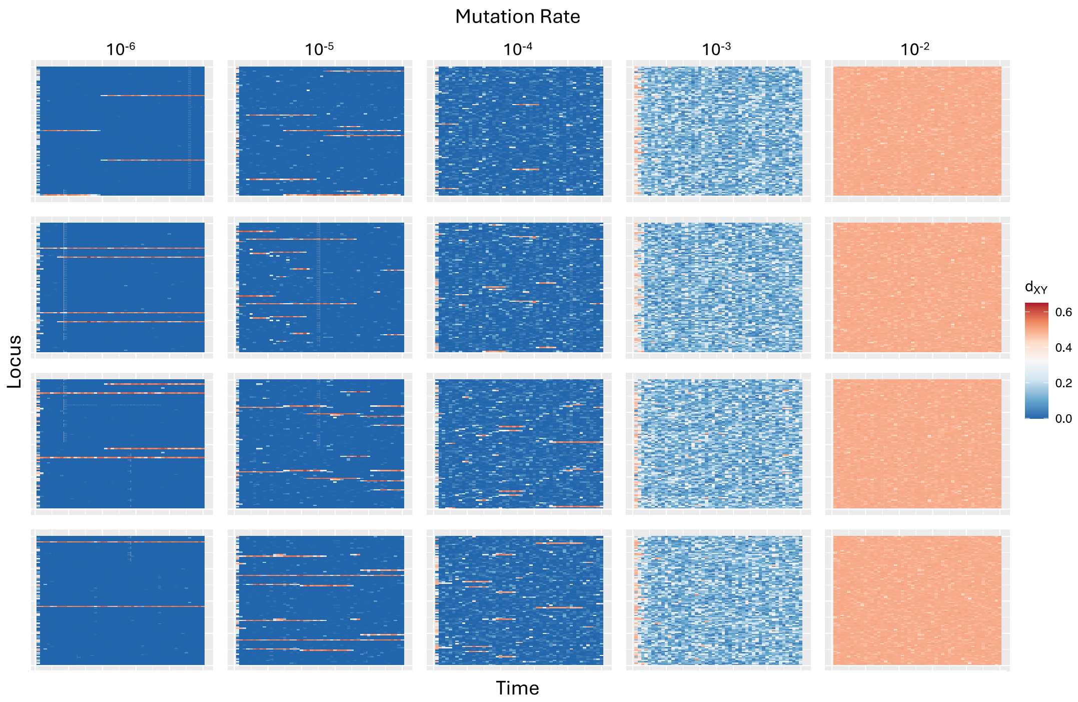

Figure S16: Effect of mutation rate, migration-selection balance, and time on sequence divergence (d_{XY}) between patches at each of one hundred genotypically redundant adaptive loci. In a single panel, the adaptive loci are shown on the y-axis and time is shown on the x-axis; each panel shows one example simulation replicate, with four replicates shown per parameter set in each column. Results are shown after 50,000 generations in 1,000 generation steps. Allele effect sizes were ± 0.25, such that an individual could reach the optimum in a patch (± 1) by being homozygous for the optimal allele at 2 loci. Each patch was comprised of N = 1,000 individuals, migration rate = $10^{-1.75}$, and V_S = 5.

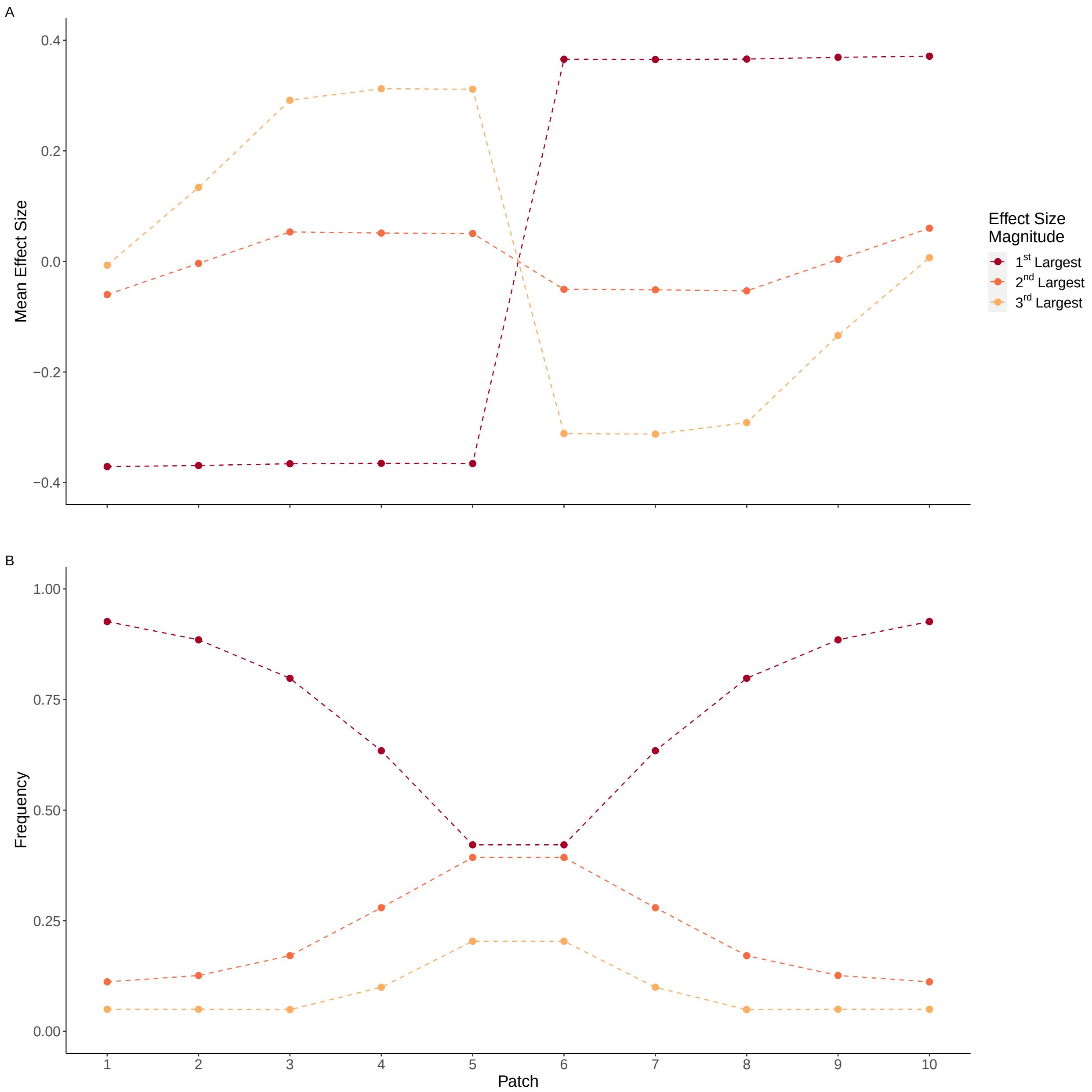

Figure S17: Effect size and frequency of the three largest magnitude alleles in each patch of a single-locus, ten-patch stepping stone model. Patch optima scaled linearly from -1 to +1 across the landscape. Results are shown after 250,000 generations. Each patch was comprised of N = 1,000 individuals, mutation rate = 10^{-5} per locus, migration rate = $10^{-1.75}$, and V_S = 5.

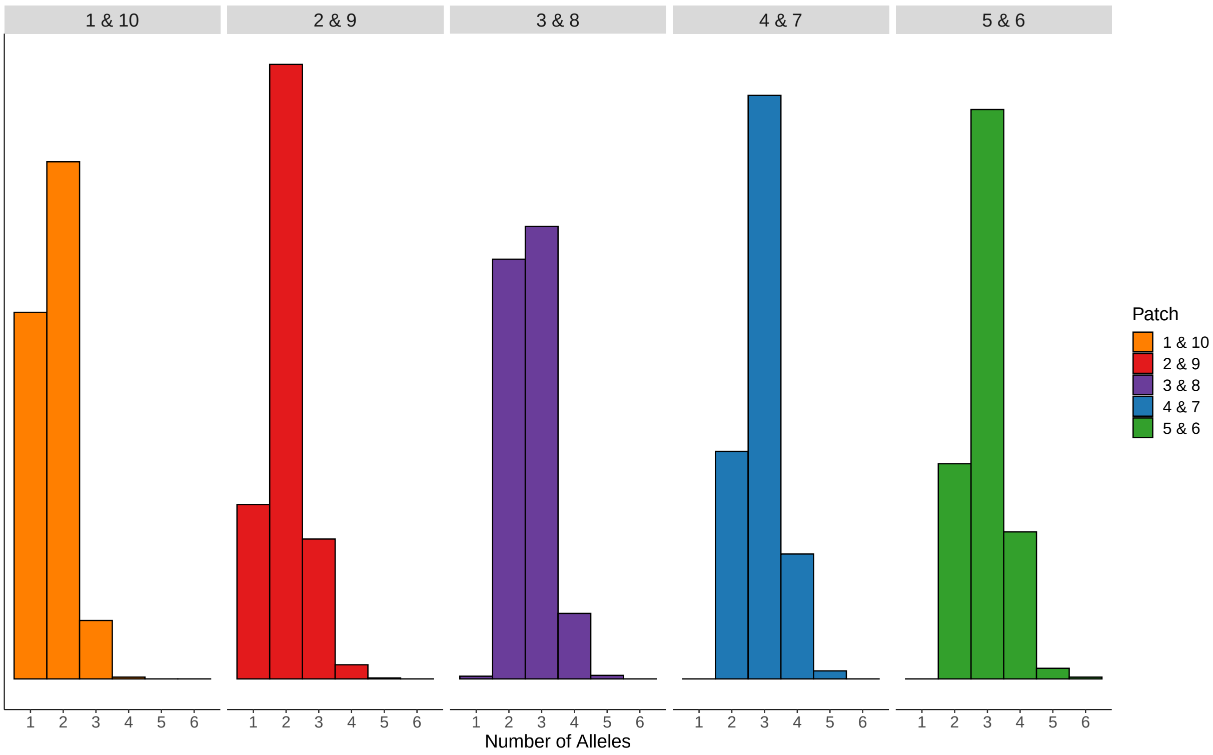

Figure S18: Number of unique alleles by patch in single-locus, ten-patch stepping stone model. A minor allele frequency threshold of 1% was applied. Results are shown after 250,000 generations. Each patch was comprised of N = 1,000 individuals, mutation rate = 10^{-5} per locus, migration rate = $10^{-1.75}$, and V_S = 5.

Supplemental References

Haller BC, Messer PW. 2019. SLiM 3: Forward genetic simulations beyond the Wright–Fisher model. Mol. Biol. Evol. 36(3):632–637.

Hudson RR. 2002. Generating samples under a Wright-Fisher neutral model of genetic variation. Bioinform. 18(2):337-338.
